# Regulation of hypothalamic reactive oxygen species and feeding behavior by phosphorylation of the beta 2 thyroid hormone receptor isoform

**DOI:** 10.1038/s41598-024-57364-9

**Published:** 2024-03-26

**Authors:** Svetlana Minakhina, Sun Young Kim, Fredric E. Wondisford

**Affiliations:** 1 Department of Medicine, Robert Wood Johnson Medical School, Rutgers, The State University of New Jersey, New Brunswick, NJ USA; 2 Mount Sinai School of Medicine, New York, NY USA; 3 University of Arizona College of Medicine, Phoenix, AZ USA

**Keywords:** Endocrine system and metabolic diseases, Cell death in the nervous system, Feeding behaviour, Molecular neuroscience

## Abstract

Unlike other thyroid hormone receptors (THRs), the beta 2 isoform (THRB2) has a restricted expression pattern and is uniquely and abundantly phosphorylated at a conserved serine residue S101 (S102 in humans). Using tagged and or phosphorylation-defective (S101A) THRB2 mutant mice, we show that THRB2 is present in a large subset of POMC neurons and mitigates ROS accumulation during ROS-triggering events, such as fasting/refeeding or high fat diet (HFD). Excessive ROS accumulation in mutant POMC neurons was accompanied by a skewed production of orexigenic/anorexigenic hormones, resulting in elevated food intake. The prolonged exposure to pathogenic hypothalamic ROS levels during HFD feeding lead to a significant loss of POMC neurons in mutant versus wild-type (WT) mice. In cultured cells, the presence of WT THRB2 isoform, but not other THRs, or THRB2^S101A^, reduced ROS accumulation upon exogenous induction of oxidative stress by tert-butyl hydroperoxide. The protective function of phospho-THRB2 (pTHRB2) did not require thyroid hormone (TH), suggesting a TH-independent role of the THRB2 isoform, and phospho-S101 in particular, in regulating oxidative stress. We propose that pTHRB2 has a fundamental role in neuronal protection against ROS cellular damage, and mitigates hypothalamic pathological changes found in diet-induced obesity.

## Introduction

Cellular actions of thyroid hormones (THs) are principally mediated by thyroid hormone receptors (THRs)^1^. Two genes, *Thra* and *Thrb* encode four major THR isoforms: THRA1 and 2, present in most tissues, and highly enriched in the brain; THRB1 also detected in multiple tissues, but dominating in the liver; and THRB2 with the most restricted pattern, detected in the anterior pituitary, hypothalamus, inner ear and retina^1–3^. Pituitary thyrotrophs express the highest levels of THRB2, easily detectible by RNA and protein analyses^3–7^. *Thrb2* mRNA has been detected in the paraventricular nucleus (PVN) and several other parts of the rodent brain, including the arcuate nucleus (Arc)^4,5,8^, but information on THRB2 protein distribution in the Arc is scarce. Isoform specific antibodies have been generated by several researchers^2,9–11^. However, none of the antibodies is currently available to the broader research community.

THRB2 contains a conserved phospho-serine residue at position 101 (pS101, pS102 in human) in the N-terminal AF-1 domain^12^. Our laboratory has shown this to be a major phosphorylation site and that other THR isoforms’ phosphorylation is not as prominent^12^. The AF-1 domain and pS101 have been implicated in TH-independent actions, integrating both TH and nonhormonal cues in response to environmental and nutritional challenges^12,13^.

One of the prominent functions of THs and their receptors is to control cellular levels of reactive oxygen species (ROS)^14–16^. However, the role of THs in maintaining cellular redox balance is complex. THs promote mitochondrial oxidative phosphorylation (OXPHOS) and regulate the TCA cycle and fatty acid catabolism, thus contributing to both increases and decreases in ROS, depending on the cellular context. THR-mediated functions also include regulation of classical antioxidant enzymes and proteins, such as superoxide dismutase, catalase, glutathione peroxidases, glutathione–S transferases, cytochrome oxidases, and uncoupling proteins 1 and 3 (UCP1 and 3)^14–20^. While THRs directly regulate many pro- and anti-oxidative genes at the transcriptional level^21,22^, the mechanism by which TH and THRs regulate cellular ROS levels remains unclear.

Accumulation of ROS makes neurons more susceptible to oxidative damage, but ROS have also attracted attention as secondary messengers in the brain^23–26^. Physiological regulation by ROS has been reported in several cell types, in particular, in proopiomelanocortin (POMC) and agouti-related protein (AgRP)/neuropeptide Y (NPY) neurons, the two major neuronal types essential for control of feeding and energy metabolism, has been investigated^24–27^. POMC neurons express several active metabolites from the POMC precursor, including anorexigenic melanocyte stimulating hormone 1 (α-MSH), and orexigenic β-endorphin (β-EP)^28–30^. Orexigenic AgRP/NPY neurons produce AgRP that acts as an inverse agonist of α-MSH receptor, MC3/4^31^. Both, POMC and AgRP/NPY neurons are sensitive to ROS regulation. Interestingly, AgRP/NPY neurons are activated during negative energy balance, with low basal ROS levels despite increased firing and substrate utilization^32^, and an increase of ROS seems to lessen their activities^26,32^. Conversely, during positive energy balance, when POMC neurons are most active, ROS accumulates in these POMC cells, suggesting that POMC activation is driven by ROS^32,33^. In both types of cells, ROS are likely to be regulated by UCP2^27,32,34,35^. POMC ROS levels and neuronal activity raise at satiety and being induced by metabolites such as glucose, lactate and fatty acids and signaling by insulin, leptin, and endocannabinoids. Importantly, these physiologically high ROS levels can be further increased, for instance, by exogenous activation of cannabinoid receptor 1 (CNR1)^27^. Pathologically high ROS levels are associated with increased production of β-EP versus α-MSH from the POMC peptide, ultimately increasing appetite. Basal ROS levels are also increased during high fat diet (HFD) feeding and may contribute to reduced sensitivity of POMC neurons to orexigenic cues^26,36,37^. Overall, POMC neurons demonstrate higher ROS production than many other neurons, including AgPR/NPY^26,32^, suggesting that POMC neurons may be particularly prone to free-radical induced dysfunction and eventual degeneration during chronic elevation of ROS caused by stress, diet-induced obesity and type 2 diabetes^26,32^.

Robust anti-oxidative mechanisms are especially important for function and survival of cells with high ROS turnover. A number of studies recognized a protective role of THs and THRs in ROS-mediated neuronal injury. The mechanisms include activating UCP2, promoting mitophagy, or modifying a chromatin landscape through histone modification and DNA methylation^35,38–41^. The individual roles of THRs isoforms in these processes are however unknown. Interestingly, THRA1/2 isoforms are present in most neurons, but THRB1/2 isoforms are significantly enriched in Arc POMC versus AgRP/NPY neurons^42^. The Arc also has the capacity for local production of the most biologically active TH, triiodothyronine (T_3_), which is found to stimulate UCP2 production in AgRP/NPY, but not in POMC neurons^35,43^, suggesting that the mechanisms regulating ROS in these types of cells are different.

In our study we aimed to elucidate the role of THRB2 pS101 phosphorylation in hypothalamic response to nutritional challenges. We described specific function of pTHRB2 in POMC neurons, focusing on its role in regulating ROS levels, and ultimate protection of POMC neurons from dysfunction and oxidative damage.

## Results

### Role of THRB2 S101 in energy metabolism and food intake

**Figure 1:**
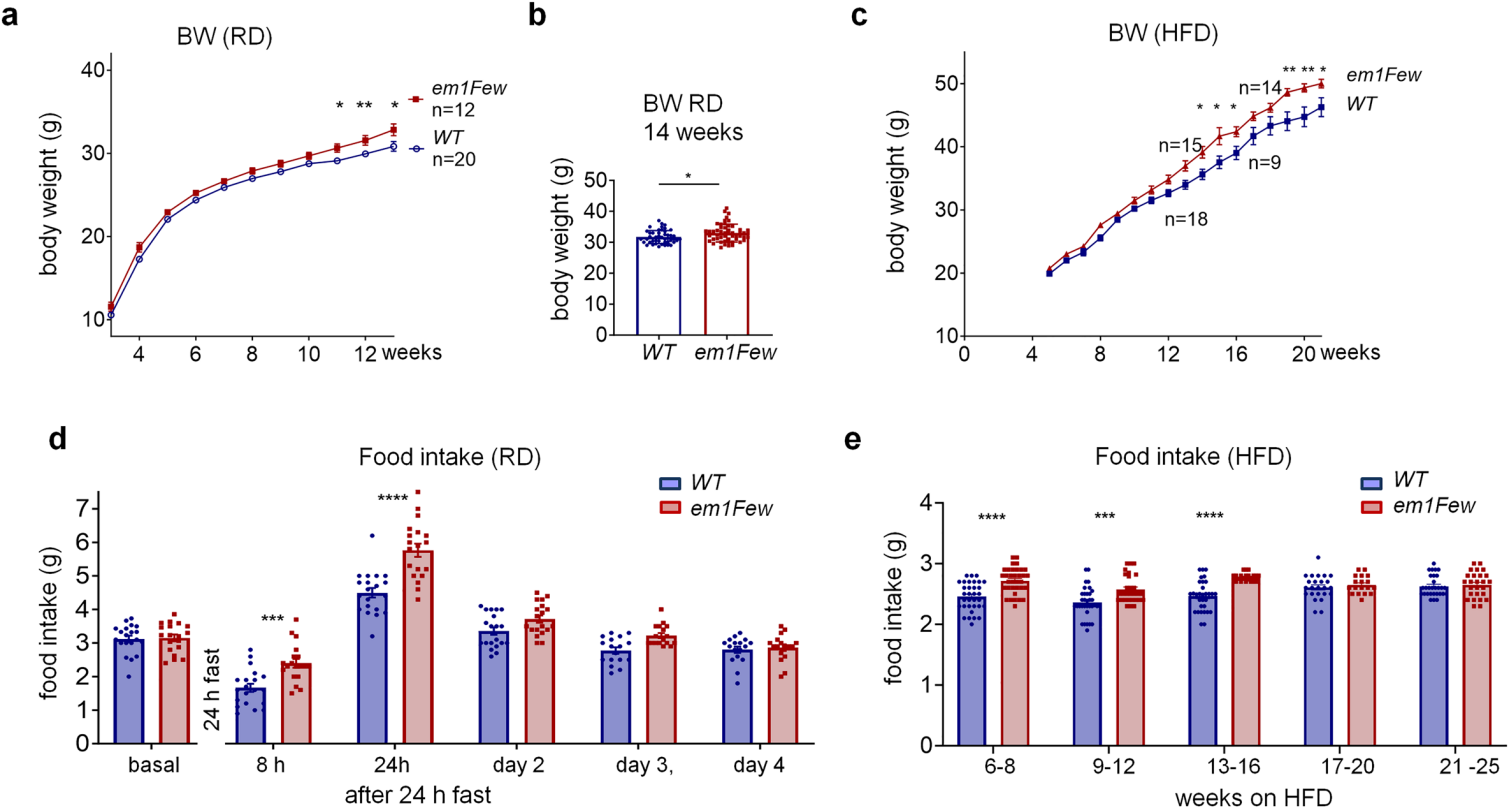
*Thrb*^*em1Few*^ animals show hyperphagia in response to nutritional challenges. (**a**) Body weight (BW) curves of *Thrb2*^*WT*^ (*WT*) and *Thrb*^*em1Few*^ (*em1Few*) mice fed a regular diet (RD). BW measurements were taken weekly. (**b**) A significant increase *Thrb*^*em1Few*^ BW at 3 months was observed (n > 50 mice). (**c**) *Thrb*^*em1Few*^ animals showed elevated BW after 8 weeks on a high-fat diet (HFD). (**d**) 24 h fasting induces temporary hyperphagia in *Thrb2*^*S101A/S101A*^ animals, evident 8 h and 24 h after fasting. (**e**) *Thrb*^*em1Few*^ animals demonstrate increased food intake up until 16 weeks on HFD. Two weeks averaged daily food intake is shown. Each dot represents a measurement from an individual mouse, values are mean +/− SEM, Two-way ANOVA and Sidak’s multiple comparison test was used in (a, c, d and e), two tailed unpaired t-test for (**b**), ****p < 0.0001, ***p < 0.001, **p < 0.01, *p < 0.05, ns for not significant, values are shown in Supplementary Table 1.

In our previous studies of THRB2 phosphorylation during caloric deprivation^12^, we observed abnormal feeding behavior in *Thrb*^*em1Few*^ (previously known as *Thrb2*^*S101A*^) homozygous mutant mice (Fig. 1). A statistically significant increase in body weight (BW) in mutant compared to the control mice was observed in the current study after feeding a regular diet (RD) (Fig. 1b, > 50 mice in each group) or high-fat diet (HFD) (Fig. 1c, > 15 mice in each group). Interestingly, basal food intake in RD fed *Thrb2*^*em1Few*^ mice was not increased compared to WT; however, mutant mice showed increased food intake within the first 24 h of refeeding after a 24 h fasting period (Fig. 1d). On subsequent days, feeding by mutant mice was no different than WT mice. Unlike RD feeding, mutant mice displayed an increase in basal food intake during the first 16 weeks of HFD feeding (Fig. 1e). Increased food intake normalized after 16 weeks of feeding (weeks 17–25, Fig. 1e).

Importantly, the S101A mutation did not affect animal activity, energy expenditure (EE), and respiratory exchange ratio (RER) under both RD and HFD conditions (Supplementary Fig. 1a and b). Fasting and refeeding also have similar effects on energy metabolism in both WT and *Thrb*^*em1Few*^ mice (Supplementary Fig. 1c). This observation agrees with the absence of significant hypo- or hyperthyroidism in mutant mice in the basal state^12^ (Supplementary Fig. 2). Our current and previous data suggest that mutant mice are defective in acute suppression of the hypothalamic-pituitary-thyroid (HPT) axis in response to caloric deprivation after 24 h fasting, but show no difference in TH levels at refeeding or HFD compared to control animals (Supplementary Fig. 2a and b).

To determine if differential sensitivity to TH could explain differences in food intake, WT and *Thrb*^*em1Few*^ mice were given low-dose T_3_ in drinking water (0.3 mg/ml) to induce hyperphagia without causing significant thyrotoxicosis (Supplementary Fig. 2c and d). Both groups showed a similar increase in food intake and body weight (Supplementary Fig. 2d and e), suggesting that THRB2 phosphorylation controls food intake independently of TH levels and its acute effects on the HPT axis. This result is interesting as Hameed et al. demonstrated that the orexigenic T_3_ effect in mice is localized to the VMH and mediated by THRB^44^.

### THRB2 expression in POMC neurons

**Figure 2:**
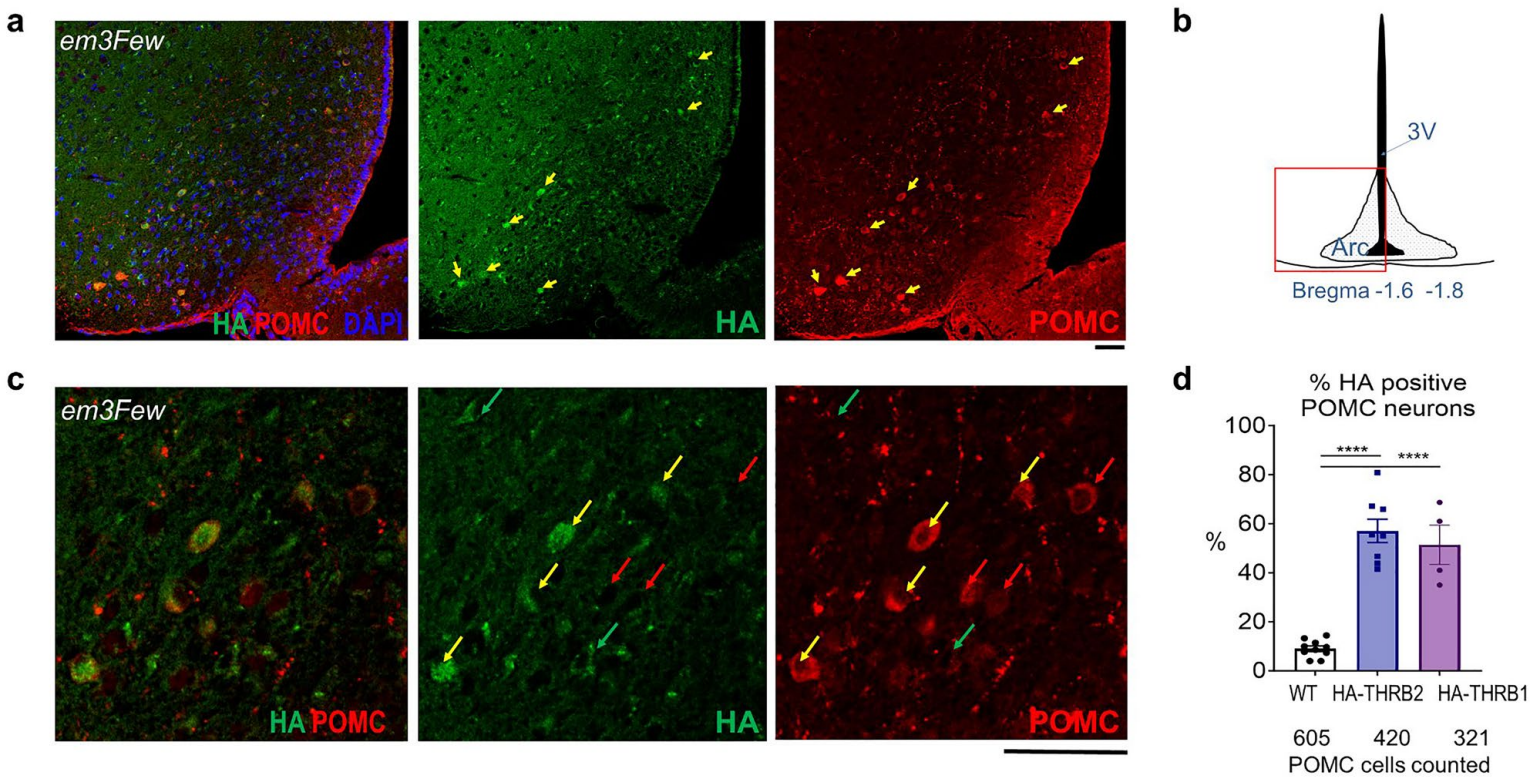
THRB2 is expressed in Arc POMC neurons. (**a**–**c**) Co-localization of POMC (red) staining and HA-immunofluorescence (green) in arcuate nucleus (Arc) of *HA-Thrb2* mice.Yellow arrows show cells co-stained with anti-HA and anti-POMC antibodies, red—POMC neurons with now or low HA, green—background staining in POMC negative cells. (**b**) Location of the hypothalamic cross section. DNA stained with DAPI (blue). Percentage of HA positive POMC neurons shown on bar graph (**d**). Each dot represents a measurement from an individual mouse, values are mean +/− SEM, One-way ANOVA and Tukey’s multiple comparison test used, ****p < 0.0001. Total number of POMC counted neurons is indicated under the graph. (**e**) Assessment of HA-antibody background staining using WT animals. Scale bar 50 μM.

THRB2 is highly expressed in pituitary thyrotrophs^3–7^, while THRB2 expression in other tissues is significantly less abundant. We used our endogenously HA (hemagglutinin)- tagged mouse line, *Thrb*^*em3Few*^ (known as *Thrb2*^*HA*^)^3^, to explore THRB2 expression pattern in the hypothalamus, the major site regulating mouse feeding behavior. WT tissues without HA expression were used as a negative controls. Two different anti-HA antibodies showed specific immunoreactivity in the PVN and Arc (Fig. 2 and Supplementary Fig. 3). The PVN is an established site of THRB2 action^45^, whereas the expression and function of THRB2 in rodent Arc is unknown.

We found that HA-positive THRB2 cells in the Arc show a high level of co-localization with POMC immunoreactivity (Fig. 2a–d). To assess the specificity and the level of co-localization, we quantified POMC cells that also contained HA immunoreactivity (Fig. 2d). The percentage of POMC cells positive for HA-THRB2 was approximately 55%, and significantly exceeded the numbers of background immunoreactivity spots seen in WT POMC neurons (< 10%). Hypothalamic background HA immunoreactivity outside of POMC neurons (Fig. 2 and Supplementary Fig. 3e, green arrows) (~ 85 positive spots/mm^2^) was somewhat lower in HA-THRB2 compared to WT mice (~ 120 spots/mm^2^), suggesting that other cell populations in Arc and the adjacent VMH were unlikely to express significant amounts of THRB2. Moreover, in our analysis of HA-THRB2 immunoreactivity, we could not find evidence of HA-THRB2 expression in AgRP neurons (Supplementary Fig. 3g).

Previously published analysis of cell-type specific transcriptomics^42^ shows significantly higher *Thrb* mRNA levels in POMC compared to AgRP/NPY cells. Although this approach did not differentiate between *Thrb1* and *Thrb2* isoforms, it confirms that *Thrb1/2* mRNA levels are found in POMC neurons. We also used an endogenously tagged HA-THRB1 (*Thrb*^*em2Few*^) mouse line^3^, and found HA-THRB1 was present in ~ 50% of POMC neurons (Fig. 2d). However, we could not assess whether the two isoforms were present in the same or different cells.

### Effect of appetite-regulating hormones on *Thrb*^*em1Few*^ animals

**Figure 3:**
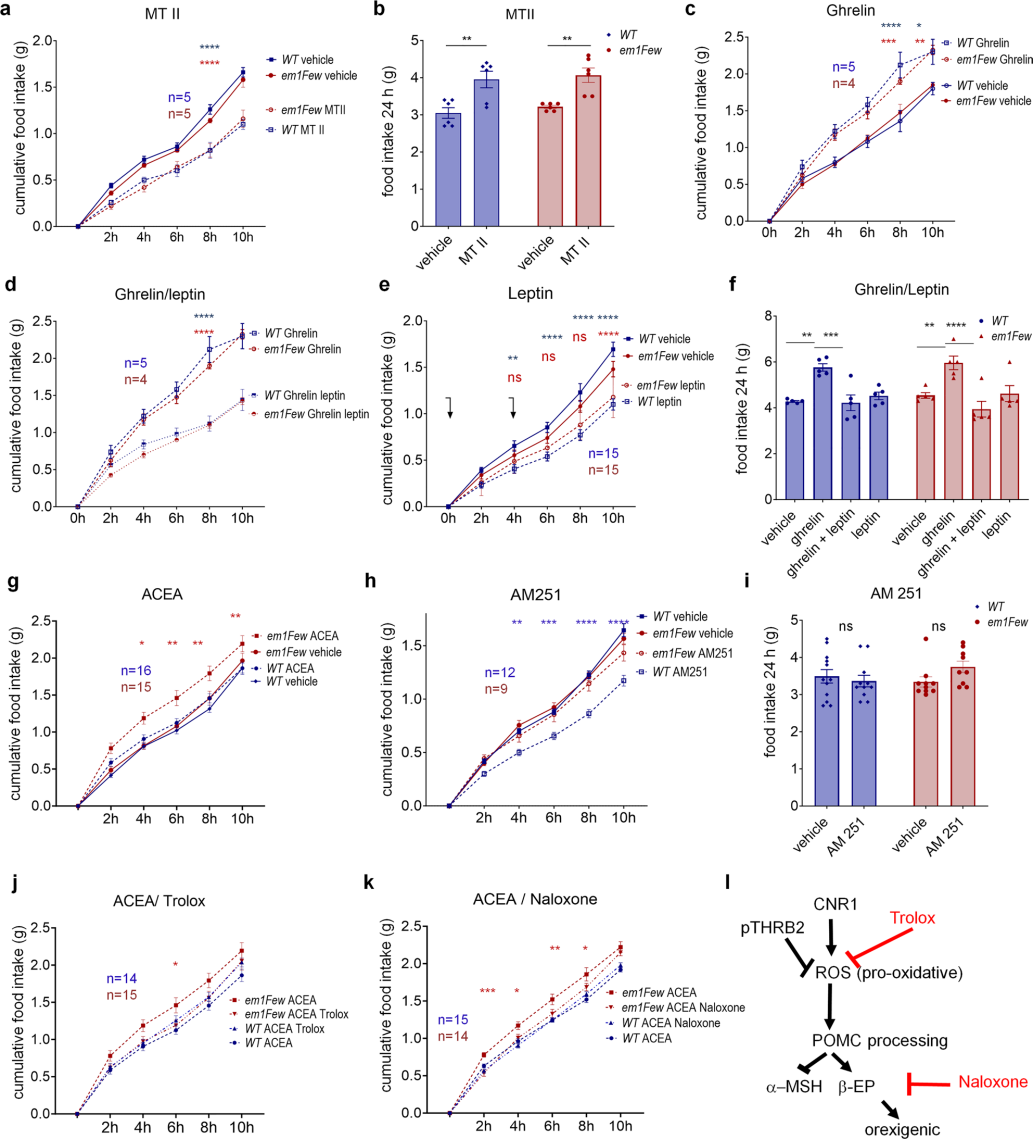
Feeding response of *Thrb*^*em1Few*^ and WT animals to administration of appetite regulators. (**a**) Cumulative food intake after 2 h fasting followed by injection (vertical arrow) of MTII (dashed lines, **a**), Ghrelin (dashed lines, **c**), Ghrelin/Leptin (**d**), ACEA (dashed lines, combined data from 2 experiments, **g**), AM251 (dashed lines, **h**). Solid lines depict food intake of the same group of animals after vehicle injection. (**e**) Leptin was administrated to free-fed animals with two injections at 0 and 4 h (combined data from 3 experiments). Food accumulation was measured every 2 h for 10 h, and total food intake 24 h after injection (**b**, **f** and **i**). ACEA/Trolox (dotted lines, **j**), ACEA/Naloxone (dotted lines, **k**) administration compared to ACEA injection (combined data from 2 experiments. Numbers of animals (n) per group is shown on each graph. All values are +/− SEM. Two way ANOVA and Tukey’s multiple comparison test used ***p < 0.001, **p < 0.01, *p < 0.05, ns for not significant (blue—between treatments of WT, red—between treatments of mutant, black between different genotypes/same treatment), point by point *p* values and comparison of Area under the curve (AUC) are available in Supplementary Tables 1 and 2. (**l**) Schematic illustration of orexigenic effects caused by CNR1 activation, and role of pTHRB2 in increased sensitivity to CNR1 stimulation.

After finding THRB2 in the Arc and PVN, we tested the function of these neurons, seeking to identify signaling pathways affected by THRB2 S101 phosphorylation. First, we used melanotan II (MTII), a MC4R agonist that targets PVN neurons, reduces the orexigenic effects of AgRP/NPY, and diminishes food intake^46–48^. Food intake in WT and mutant animals was synchronized by a short fasting period (2 h) that did not cause a difference in feeding behavior between genotypes (see vehicle injection in Fig. 3a–h, solid lines). Single I.P. injections of MTII significantly decreased food intake up to 10 h after injection (Fig. 3a). At 24 h, the anorexigenic effect decreased and animals showed an increase in total food intake, compensating for the period of underfeeding (Fig. 3b). Both effects were very similar in control and mutant animals, ruling out MC4R as a direct effector or regulator of pTHRB2.

Another group of animals was given ghrelin, which targets AgRP/NPY neurons, and causes a strong increase in food intake^48,49^. Ghrelin had indistinguishable orexigenic effects on WT and *Thrb*^*em1Few*^ animals, observed during the first 24 h (Fig. 3c and f), and in both genotypes, this effect was inhibited by leptin (Fig. 3d and f). The result was not surprising, because AgRP/NPY neurons are dominant regulators of the orexigenic response^49^, and THRB2 is not found in most AgRP/NPY neurons (Supplementary Fig. 3g). Interestingly, the single injection of leptin alone did not cause a reduction in food intake in either genotype, but two consecutive injections of leptin in sated mice caused the expected anorexigenic effect in WT (Fig. 3e), while the effect in *Thrb*^*em1Few*^ mice was much less (also see Supplementary Tables 1 and 2). This phenotype suggests the potential of a pTHRB2 interaction with leptin pathway in POMC and/or PVN neurons.

To specifically test the function of POMC neurons in *Thrb*^*em1Few*^ mice, we probed the CNR1 signaling pathway^27,42,49^. Studies by Koch and colleagues^27^ indicate that exogenous CNR1 activation causes elevated levels of ROS in POMC neurons, leading to unbalanced POMC processing, and elevated levels of β-EP. We utilized a CNR1 selective agonist (arachidonyl-2-chloroethylamide, ACEA) and an antagonist (AM251) in WT and *Thrb*^*em1Few*^ animals. ACEA injection caused an increase in food intake in *Thrb*^*em1Few*^ mice compared to vehicle or WT control mice (Fig. 3g). Conversely, AM251 injection (0.6 mg/kg) caused a significant reduction in food intake in WT but not *Thrb*^*em1Few*^ mice (Fig. 3h and i). In contrast, administration of a higher dose of AM251 (2 mg/kg) was able to inhibit food intake in both WT and *Thrb*^*em1Few*^ mice (Supplementary Fig. 4a), suggesting that S101A mutation caused an increased sensitivity of POMC neurons to cannabinoids.

To test if the ACEA-induced hyperphagia in *Thrb*^*em1Few*^ mutant mice was due to increased ROS levels and/or elevated β-EP, we combined CNR1 activation with: (i) an antioxidant (Trolox) to reduce ROS levels, or (ii) an opioid antagonist (naloxone) to block β-EP (Fig. 3j–l). Importantly, both Trolox and naloxone were able to suppress excessive food intake in *Thrb*^*em1Few*^ animals (Fig. 3j–l). Therefore, pTHRB2 may have an important function in downregulating cellular ROS, protecting POMC neurons from signal-induced oxidative stress, and abnormal POMC processing.

### pTHRB2 regulates ROS in POMC neurons in response to a nutritional challenge

**Figure 4:**
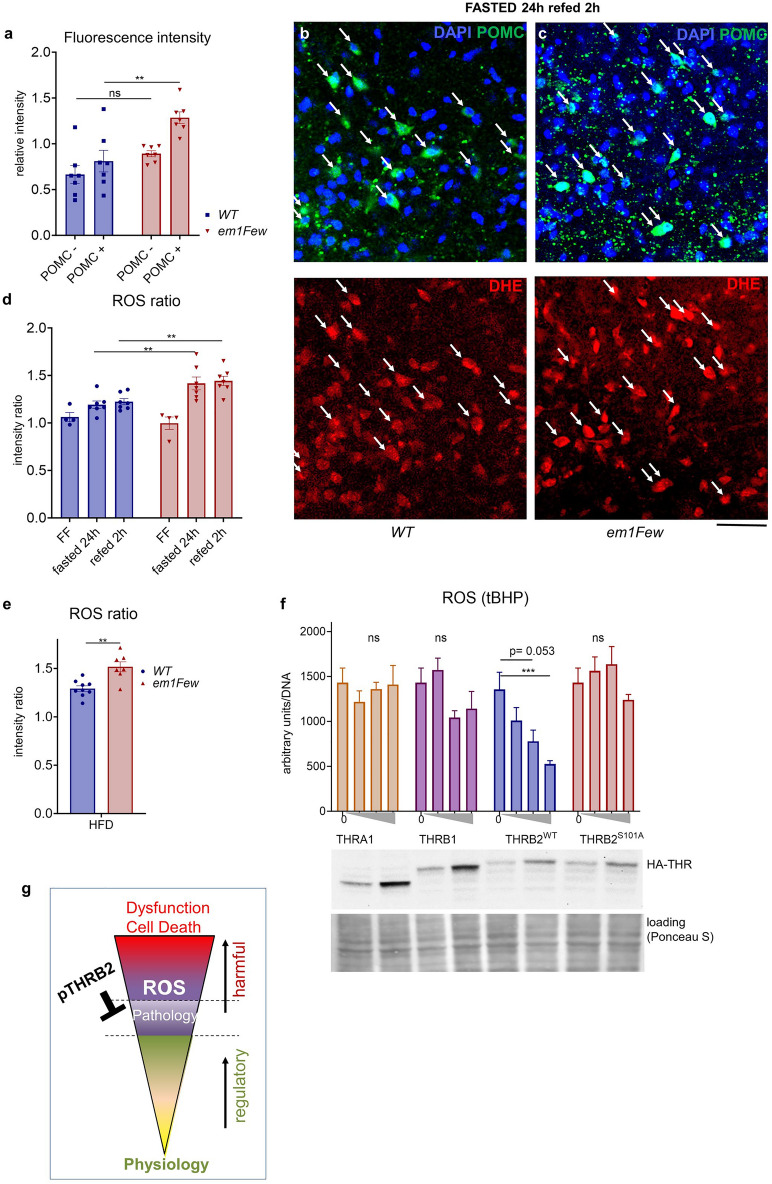
Lack of THRB2 phosphorylation (*Thrb*^*em1Few*^) causes increased ROS accumulation in POMC neurons and cultured neuroblastoma cells. (**a**) DHE fluorescence intensity, reflecting ROS levels, in POMC− and POMC + Arc neurons in refed state (based on anti-POMC antibody immunostaining) (**b** and **c**) POMC (green), DHE (red) Arc neurons (arrows) of the indicated genotypes. DNA stained with DAPI (blue). Scale bar 50 μM. (**d**) The POMC/Arc ROS ratio (ROS in POMC+/ROS in POMC− in the Arc) in free-fed (FF), fasted and refed states on RD, and (**e**) on HFD. Each dot represents the average measurement from > 200 cells in individual mice. (**f**) Expression of incrementing amounts of HA-THRB2^WT^ reduces ROS accumulation in tBHP (75 μM, 2 h) treated Neuro-2a cells. HA-THRA1, HA-THRB1 and HA-THRB2^S101A^ expressed at similar levels have no significant effect on ROS accumulation (n > 4). Western blot shows the expression of HA-THRs at the two highest concentrations, Ponceau S used as a loading control (full images available in Supplementary Fig. 6). All values are +/− SEM, two way ANOVA and Tukey’s multiple comparison test used, ****p < 0.0001, ***p < 0.001, **p < 0.01, *p < 0.05, ns for not significant, numerical p-values are available in Supplementary Table 1. (**g**) Schematic representation of ROS effects at different levels, from regulatory/secondary messenger function (yellow-green) to high level (red) damaging effects ultimately leading to cell death.

To test if the fasting/refeeding hyperphagia of *Thrb*^*em1Few*^ mutants was linked to ROS levels in POMC neurons, we assessed ROS levels in mutant and WT animals in FF, fasted, and refed states. We injected dihydroethidium (DHE) through tail vein, and co-stained brain sections with an anti-POMC antibody (Fig. 4 and Supplementary Fig. 5). *Thrb*^*em1Few*^ mutants demonstrated a significant increase of fluorescence intensity, reflecting ROS levels, in POMC neurons after a 24 h fast followed by a 2 h refeeding (Fig. 4a–c, white arrows). However, we observed variability in fluorescence intensity from animal to animal (Supplementary Fig. 4b). To circumvent this problem, we determined the ratio of fluorescent intensity in POMC + versus POMC- cells in the same Arc section, which we termed the ROS ratio (Fig. 4d). The ROS ratio was significantly elevated in mutant animals after prolonged fasting (24 h) and after refeeding (Fig. 4d). Importantly, POMC neuron numbers was not affected in mutant animals fed a RD for < 14 weeks (Supplementary Fig. 4c). Like fasting and refeeding, *Thrb*^*em1Few*^ mutant mice on a HFD also demonstrated an increase in the ROS ratio (Fig. 4e), suggesting that pTHRB2 may provide cell-specific protection against oxidative stress induced by various nutritional challenges.

### pTHRB2 regulates ROS in neuroblastoma cell lines

To test the direct role of THRB2 phosphorylation in ROS metabolism, we employed two neuroblastoma cell lines, Neuro-2a and N1E-115 (commercially available, see Methods). Both lines express *Thra*, but have extremely low or no *Thrb1/Thrb2* expression, allowing us to develop a neuronal cell model expressing similar levels of HA tagged THRB1, THRB2, and THRB2^S101A^^50^. Cells expressing incrementally higher levels of THRs were subjected to oxidative stress using tBHP (Fig. 4f and Supplementary Figs. 5b, c and 6). ROS levels were measured using CellROX™ green reagent. We found that expression of WT THRB2 significantly decreased intracellular ROS accumulation in both cell lines in a concentration-dependent manner, while THRA, THRB1 or THRB2^S101A^ did not show significant antioxidative properties when expressed at similar levels (Fig. 4f and Supplementary Figs. 5b, and 6). THRB2 S101 is a target of CDK2 phosphorylation^12^, and treatment with CDK inhibitors (NU6027 and flavopiridol) abolished the protective effect of THRB2, confirming that S101 phosphorylation was indeed required for antioxidative function of THRB2 (Supplementary Fig. 5d and e).

### The *Thrb*^*em1Few*^ mutation affects POMC neuronal function

**Figure 5:**
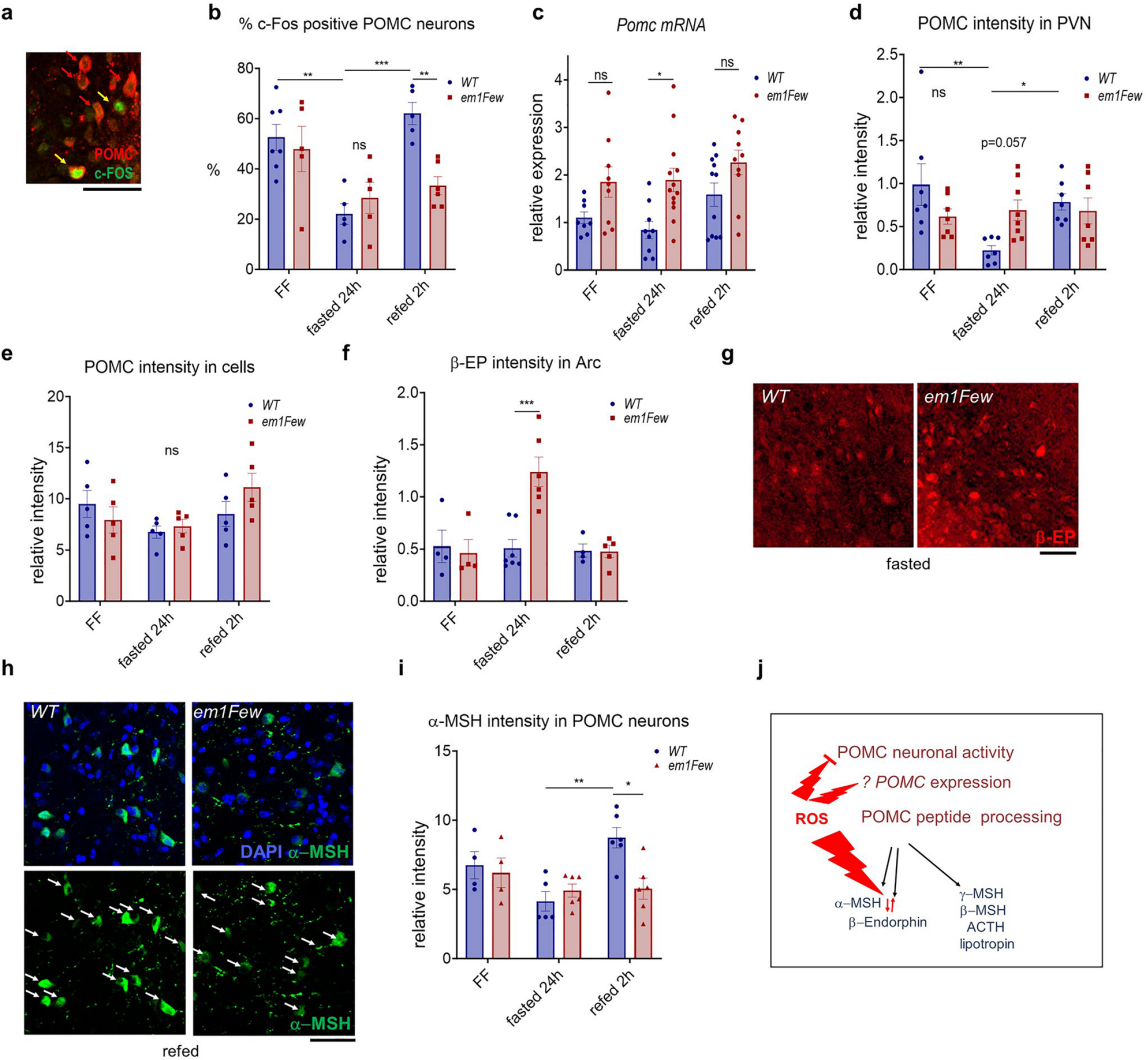
*S101A* mutation differentially affects POMC, α-MSH and β-EP production in POMC neurons in response to fasting/refeeding. (**a**, **b**) Percentage of c-FOS (green) positive POMC (red) neurons in WT and S101 phosphorylation defective *Thrb*^*em1Few*^ (*em1Few*) animals in free-fed (FF), fasted, and refed states. *Pomc* mRNA levels in mediobasal hypothalamus (**c**), POMC relative fluorescent intensity in PVN (**d**), and (**e**) in POMC Arc neurons. (**f**, **g**) β-EP (red) relative intensity in the POMC neurons and Arc. (**h**, **i**) α-MSH (green) relative intensity within POMC neurons (arrows). DNA stained with DAPI (blue). Scale bar 50 μM. Additional images are available on Supplementary Figs. 7 and 8. Each dot on the graphs represents the mean measurement (> 100 cells per animal) from individual mice; all values are +/− SEM, two way ANOVA and Sidak’s multiple comparison test used, numerical p-values are available in Supplementary Table 1, ***p < 0.001, **p < 0.01, *p < 0.05, ns for not significant. (**j**) Model of pathogenic ROS effects on neuronal activity, POMC levels and processing.

Low or physiological ROS levels have regulatory functions in Arc neurons. However, oxidative stress may dysregulate both neuronal activity and hormone production^26,27,34,37^. To assess POMC neuronal activity in the Arc of WT and *Thrb*^*em1Few*^ animals, we stained POMC cells in the free-fed, fasted and refed states with an anti c-FOS antibody (Fig. 5a and Supplementary Fig. 8a). As expected in WT animals, the percentage of c-FOS positive POMC neurons fell significantly after 24 h fasting, and increased ~ 3 fold upon refeeding. In contrast, the activity of POMC neurons in *Thrb*^*em1Few*^ mutants did not show a significant increase upon refeeding (Fig. 5b).

Abnormal ROS levels affect the production of POMC-derived hormones^27^. We found that POMC expression and peptide processing were disturbed in *Thrb*^*em1Few*^ animals. First, after 24 h fasting, *Pomc* mRNA levels in mediobasal hypothalamus of *Thrb*^*em1Few*^ mutants were significantly higher than in controls mice (Fig. 5c). During fasting refeeding WT animals showed no visible changes in POMC intensity in POMC neurons but demonstrated a significant reduction of POMC immunoreactivity in PVN, compared to that in free-fed and refed states (Fig. 5d, e, Supplementary Fig. 7b and f). While POMC intensity in mutant POMC neurons were not different from that in WT, the down-regulation of POMC in PVN projections at fasting was abolished (Fig. 5d, e, Supplementary Fig. 7b and f). POMC level abnormalities were accompanied by skewed production of β-EP and α-MSH. At fasting mutant animals showed higher β-EP levels (Fig. 5f and g) while α-MSH levels were similar to that in control (Fig. 5h and i). At refeeding, when POMC and β-EP levels in WT and mutants were similar, α-MSH intensity in mutant POMC neurons was significantly lower (Fig. 5d–f, h, i and Supplementary Fig. 7) suggesting abnormalities in POMC peptide processing (Fig. 5j).

**Figure 6:**
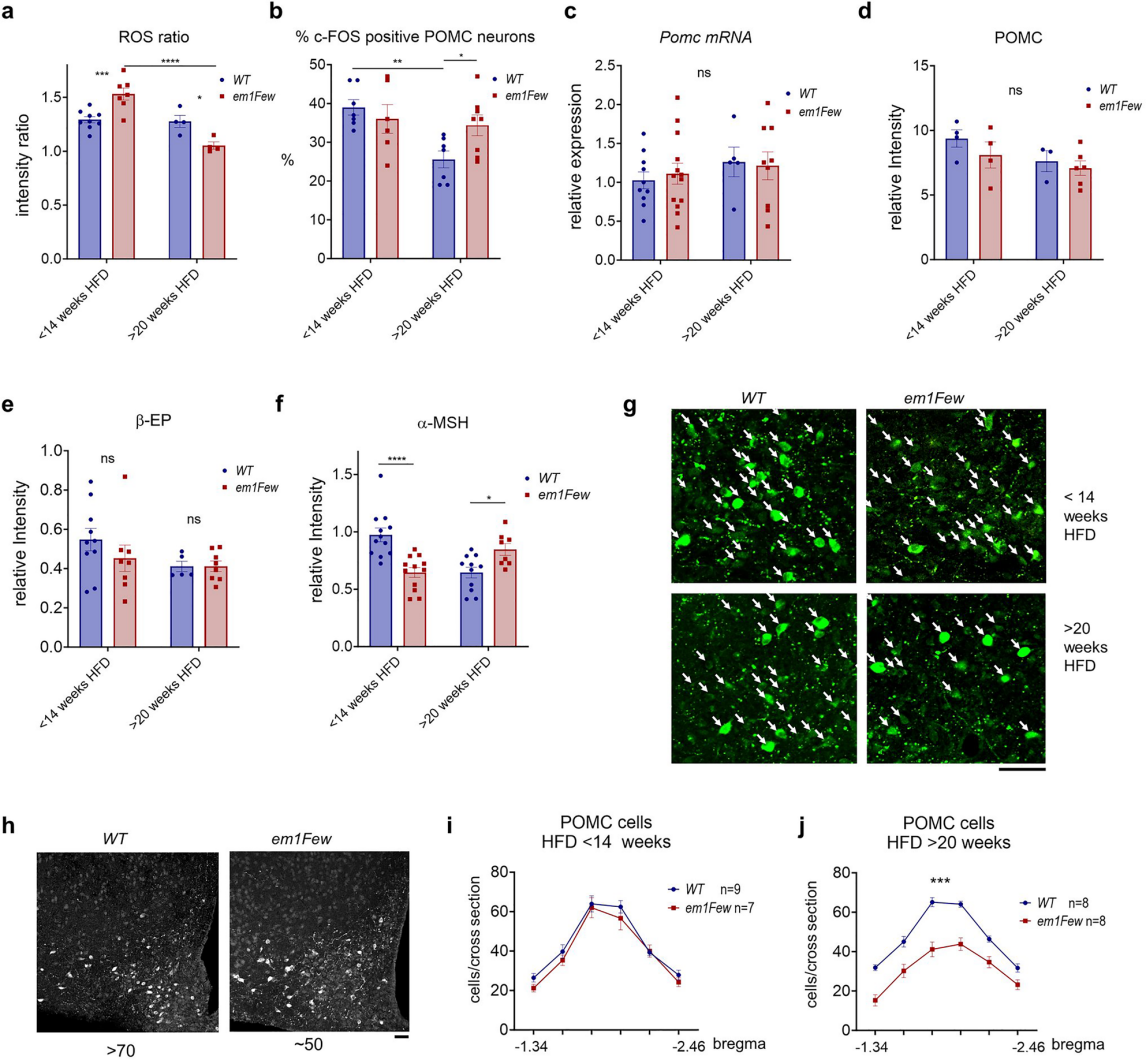
HFD causes an abnormal ROS accumulation in *Thrb*^*em1Few*^ POMC neurons, skewed α-MSH and β-EP production, and eventual neuronal loss. (**a**) POMC+/POMC− ROS ratio elevated in HFD animals (See Fig. 4e) was further increased in *Thrb*^*em1Few*^ (*em1Few*) animals, and decreased after 20 weeks on HFD. (**b**) Percentage of c-FOS-positive POMC neurons in WT and *Thrb*^*em1Few*^ animals < 14 and > 20 weeks on HFD. *Pomc* RNA levels in mediobasal hypothalamus (**c**) and POMC relative intensity in POMC neurons (**d**). (**e**) β-EP (red) relative intensity in POMC neurons. (**f**, **g**) α-MSH (green) relative intensity within POMC neurons (arrows). Scale bar 50 μM. DNA stained with DAPI (blue). Each dot on the graphs represents the mean measurement (> 200 cells per animal) from individual mice, all values are +/− SEM, two-way ANOVA and Sidak’s multiple comparison test used, ****p < 0.0001, **p < 0.01, *p < 0.05, ns for not significant. Additional images are available on Supplementary Fig. 8b, and c. (**h**) Representative cross sections of Arc with the highest counts of POMC neurons in WT and *Thrb*^*em1Few*^ animals 22 weeks on HFD. Scale bar 50 μM. (**i**, **j**) Distribution of POMC neurons throughout Arc (bregma − 1.34 to − 2.46) in WT and *Thrb*^*em1Few*^ animals < 14 (**i**) and > 20 (**j**) weeks on HFD. Average number from 3 to 4 cross sections per position per animal, animal numbers indicated on the graphs. Area under the curve (AUC) and unpaired t test was used for comparison, ***p < 0.001. Numerical *p* values are available in Supplementary Table 2.

Interestingly, *Thrb*^*em1Few*^ animals on HFD showed phenotypes similar to that observed in refed state (Fig. 6e, f, Supplementary Figs. 7 and 8), i.e. unchanged POMC and β-EP levels and a significant reduction of α-MSH. We did not detect a significant decrease in POMC neuronal activity in mutants, but that may be due to the high variability often seen in free-fed animals (Fig. 6b). Importantly, in all three cases, 24 h fasting, refeeding, and HFD, *Thrb*^*em1Few*^ animals showed an increased ROS ratio and a skewed α-MSH/β-EP production from the POMC peptide.

### Prolonged exposure to pathogenic ROS levels lead to POMC neuronal loss in *Thrb*^*em1Few*^ mutant animals

Since pTHRB2 appears to protect POMC neurons from oxidative damage (Fig. 4), we hypothesize that a lack of S101 phosphorylation after prolonged exposure to ROS inducers (i.e. HFD, and multiple fasting and refeeding cycles) may lead to cell death. The *Thrb*^*em1Few*^ animals showed a significant increase in food intake on a HFD for the first 14 weeks on the diet (Fig. 1e). During this time, the number and distribution of Arc POMC in *Thrb2*^*S101A*^ mice was similar to that in WT (Fig. 6i). A change in feeding behavior in *Thrb*^*em1Few*^ mice was attributed to increased ROS and decreased α-MSH levels in POMC neurons (Fig. 6a and f). In contrast, after 18–20 weeks on HFD *Thrb*^*em1Few*^ mutant animals showed a significant reduction in the number of Arc POMC neurons (Fig. 6h–j). At this time, very few cells with high ROS were observed in *Thrb*^*em1Few*^ POMC neurons, and the ROS ratio in mutant mice became lower than that in WT (Fig. 6a, and Supplementary Fig. 8c). The remaining POMC neurons in mutant mice had overall higher activity (as indicated by c-FOS percentage, Fig. 6b) and α-MSH levels (Fig. 6f-g), potentially compensating for their reduced numbers.

The chronic ROS increase in *Thrb*^*em1Few*^ mice caused POMC neuron loss over the period of several weeks. This process was too slow, however, and we failed to detect significant changes in cell death markers (cleaved Caspase 3 or DNA damage). To test the direct role of pTHRB2 in ROS-induced cell death, we used Neuro-2a cells subjected to acute oxidative stress. The treatment with increasing concentrations of tBHP (Supplementary Fig. 9a) caused a significant reduction of mitochondrial membrane potential, an early marker of cell death in cells expressing no THRs, THRA1, THRB1 and THRB2^S101A^. Importantly, expression of WT THRB2 protected Neuro-2a cells from the effects of these sub-lethal tBHP concentrations.

## Discussion

While pathological ROS levels can damage DNA, proteins and lipids and lead to cell death, physiological ROS levels have a vital role in cellular function^23–26,33,37,42,49^. Elevated ROS levels are associated with multiple cellular pathologies, involved in cancer, inflammation, autoimmune diseases and diabetes^15,25,51,52^. To protect against cell damage and ultimately cell death, cells with physiologically high ROS levels, such as POMC neurons, need to employ additional safeguarding mechanisms (Fig. 4g). We found that THRB2 phosphorylation protects cultured neuroblastoma cells from ROS accumulation after treatment with tBHP, suggesting that this function is cell-autonomous and TH-independent. Similarly, pTHRB2 was required to keep ROS within the normal range in POMC neurons after a nutritional challenge because lack of S101 phosphorylation in *Thrb*^*em1Few*^ animals lead to ROS accumulation.

Transient exposure to elevated ROS disrupted neuronal activity and POMC peptide processing, resulting in short-term hyperphagia lasting approximately 24 h after a fasting/refeeding challenge (Fig. 1d). Episodic increases in food intake may have contributed to the increased body weight we originally observed in RD fed mutant mice (Fig. 1a and b). The phenotype appear relatively week in low-stress laboratory conditions, but it may heighten under environmental or nutritional challenge. For instance, feeding mice a HFD had chronic effects on ROS levels, POMC processing, food intake, and body weight (Figs. 1c, e, and 6). Given the damaging properties of high ROS levels, it is not surprising that long-term, high-level ROS exposure in mutant mice caused a loss of POMC-expressing neurons (Fig. 6h–j). Importantly, the remaining POMC neurons showed a somewhat reduced ROS ratio (Fig. 6a and Supplementary Fig. 8c). Several explanations for this observation are possible: (1) the remaining POMC cell population may have had inherently lower ROS levels; (2) the remaining POMC cell population employ an alternative to THRB2 for protection from pathogenic ROS; (3) the surviving neurons in mutant mice express higher levels of other THR isoforms, compensating for intrinsically lower protective properties of these proteins (see Fig. 4f). Importantly, the surviving neurons had higher production of α-MSH, and higher neuronal activity than average POMC neurons in WT (Fig. 6b and f), that explains why animals with reduced numbers of POMC neurons show no hyperphagia compared to WT mice (see weeks 17–25 on Fig. 1e).

While reduced α-MSH and low neuronal activity provided enough reason for hyperphagia, another explanation for the observed phenotypes may lay in changed balance between α-MSH and β-EP. The skewed balance between these hormones is associated with hyperphagia^27^, and occurred in response to CNR1 stimulation. Importantly, *Thrb*^*em1Few*^ mice are more sensitive to CNR1 activation, while blocking of β-EP with naloxone suppresses the increased food intake found in mutant mice (Fig. 3g-l). Elevated β-EP with preserved α-MSH levels was found in mutant animals after prolonged fasting (Fig. 5f-i), resulting in a lower α-MSH/β-EP ratio. Two hours after refeeding, a lower α-MSH/β-EP ratio was also found in mutant mice due to lower α-MSH levels. Similarly, during HFD feeding of less than 14 weeks, mutant animals displayed reduced α-MSH levels with preserved β-EP levels (Fig. 6c-g). However, after more than 18 weeks on a HFD, when POMC neuronal loss was observed, β-EP levels in mutant animals normalized, but α-MSH levels in POMC cells were higher than WT mice (Fig. 6f). Therefore, the balance between α-MSH and β-EP may be essential to control food intake, and elevated (pathological) ROS levels cause a disruption of this fine-tuned equilibrium.

Interestingly, the numbers of POMC neurons in 3–4 month-old mice were not affected by the S101A mutation (*Thrb*^*em1Few*^) compared to WT animals (Fig. 6i and Supplementary Fig. 4c). However, after 18–20 weeks of HFD feeding a significant reduction in neuron numbers was observed (Fig. 6j). Overall, nutritionally induced neuronal loss in vivo was very slow*,* therefore we used cultured cells subjected to acute sub lethal oxidative stress. The reduction of mitochondrial membrane potential, an early marker of cell death, was observed in cultured cells expressing THRA1, THRB1 and THRB2^S101A^, but not in the cells expressing WT THRB2 (Supplementary Fig. 9a), which correlated with reduced ROS accumulation in these cells (Fig. 4f), and further confirmed the role of pTHRB2 in protection from oxidative damage.

Logically, a reduced number of POMC neurons in *Thrb*^*em1Few*^ animals should have caused increased food intake. However, the hypothalamic resistance found in HFD feed animals^15,34,36^ may have blunted any further increase in food intake. At the same time, the orexigenic effect of pathological ROS and a skewed α-MSH/β-EP ratio in *Thrb*^*em1Few*^ mutants would have weakened with the loss of POMC neurons, leading to eventual convergence of WT and mutant food intake (Fig. 1e).

In addition to abnormal POMC processing and a skewed α-MSH/β-EP ratio, elevated ROS levels were associated with other phenotypes in *Thrb*^*em1Few*^ mutants: (1) reduced neuronal activity at 2 h refeeding, and non-suppressed POMC mRNA levels after 24 h fasting (Fig. 5b and c). Since these phenotypic changes were not universal for all ROS-inducing conditions, they are likely indirect or systemic effects of the THRB2 mutation, for example, as a result of the impaired HPT axis after 24 h of fasting (Supplementary Fig. 2a,^12^). While elevated hypothalamic TH levels at fasting may be responsible for altered POMC expression in vivo^43,53^, pTHRB2 did not show any T_3_—specific effect on *POMC* transcription in cultured cells (Supplementary Fig. 9c). Neither had we observed THRB2-dependent regulation of *UCP2* (Supplementary Fig. 9d)*,* ruling out some simple explanations of observed phenotypes based on the regulation of one target transcript.

The full mechanism of ROS regulation by THRB2 phosphorylation has yet to be elucidated. Nonetheless, we showed that phosphorylated THRB2 mitigated nutritionally induced oxidative stress in sub-population of hypothalamic neurons, and protected against HFD-induced POMC neuronal loss in a TH-independent manner, opening new doors for therapeutic approaches in the treatment of metabolic and perhaps other neurodegenerative conditions.

## Methods

### Cell culture

N1E-115 (Cat# CRL-2263, RRID:CVCL_0451, ATCC, VA USA) and Neuro-2a cells (Cat# CCL-131, RRID:CVCL_0470, ATCC, VA, USA) were plated in DMEM (Dulbecco's Modified Eagle Medium) high glucose (Corning Cellgro, VA, USA) containing 6% fetal bovine serum (FBS, Sigma-Aldrich, MO, USA) and 1% Antibiotic—Antimycotic (Invitrogen, CA, USA). Cells were maintained at 37 °C, 5% CO_2_. Cells were transduced with replication incompetent adenoviral constructs (expressing GFP, HA-THRB2^WT^, HA-THRB2^S101A^, HA-THRB1, and HA-THRA1*)* as described previously^12,50,54^. Treatments with T_3_, tert-Butyl Hydroperoxide (tBHP, Thermo Fisher Scientific, PA, USA), were performed in DMEM high glucose containing 6% charcoal:dextran stripped FBS (Gemini bio-products, CA, USA) 24 h after transduction.

### Reactive oxygen species and mitochondrial membrane potential measurements

Cells were seeded into black 96-well optical bottom cell culture plates (10,000 cells/well) 2 days before treatments, and 24 h before treatments, cells were transduced with *HA-Thr(s)* adenoviral constructs. Treatments with Flavopiridol and NU6027 (REF, 100uM, Selleckchem, Thermo Fisher Scientific, PA, USA) were performed in DMEM high glucose containing 6% charcoal/dextran stripped FBS (Gemini bio-products, CA, USA). After one hour the indicted amount of tert-Butyl Hydroperoxide (tBHP) was added, in 70 min, CellROX™ Green (Thermo Fisher Scientific, PA, USA) was added to the media and incubated for additional 50 min. Cellular monolayers were rinsed once with DPBS solution (Sigma-Aldrich, MO, USA) and fixed with 2% formaldehyde, followed by measurements of fluorescence (485/520 nm). Fluorescence measurements were recorded using the Synergy (Biotek, Agilent Technology, CA, USA) microplate reader. To normalize Fluorescence intensity to cell number/DNA content, cells were further stained for DNA with Hoechst 33342 and Fluorescence was measured at (390/420 nm). For mitochondrial membrane potential measurement, MitoTracker™ Red CMXRos (570/600) was used with similar set up.

### Western blotting

Cell/tissue lysates and protein extracts were separated using gradient (4–20%) Mini-Protean TGX precast gels (Bio-Rad Laboratories, Inc.; Hercules, CA, USA). Samples were transferred onto Nitrocellulose 0.2 μm membranes (Bio-Rad Laboratories, Inc.; CA, USA), stained with Ponceau S (Fisher Scientific; NH, USA), washed in PBST (PBS (0.1 M Phosphate buffer saline, pH 7.4) with 0.2% Tween 20). Membranes were rinsed and blocked for 2 h at room temperature in 2% nonfat dry milk in PBST and probed with rabbit anti-HA (1:2000 dilution, 4970, Sell Signaling, MA, USA), and subsequently with anti-Rabbit-peroxidase-conjugated antibody (1:4000, Sell Signaling, MA, USA). Blots were washed in PBST 3 times and imaged at ChemiDoc Touch Imaging System (Bio-Rad Laboratories, Inc.; CA, USA). ImageLab 6 (Bio-Rad Laboratories, Inc.; CA, USA) was used to process images.

### RNA extraction and qRT-PCR

RNA was extracted using TRIzol Plus (Invitrogen, CA, USA), and cDNA was synthesized using the iScript cDNA Synthesis Kit (Bio-Rad, CA, USA). Real-time PCR analyses were performed using iTaq Universal SYBR Green Supermix in CFX96 Real-Time PCR detection system (Bio-Rad, CA, USA) according to the recommendations of the manufacturer. Relative mRNA levels were determined using the 2^−ΔΔCt^ method. Primer efficiencies and the PCR products were assessed by relative standard curves and melting curves. Primer sequences are listed below. The representative experiments were analyzed using a two-way ANOVA and Tukey’s multiple comparison test (GraphPad Prism, CA, USA). Statistical significance was accepted at *p* < 0.05.

#### qRT-PCR primers

*Thrb* forward 5′AACCAGTGCCAGGAATGTCG3′, reverse 5′CTCTTCTCACGGTTCTCCTC3′, *Thrb2* forward 5′TGTATGCTCTCCGAGTATATGCAC3′ and reverse 5′CCATGTCCAAGTCAGAGTCCTTG3′ *Rpl13a* forward 5′GCTGCTCTCAAGGTTGTTCG3′, reverse 5′CCTTTTCCTTCCGTTTCTCC3′, *Pomc* forward 5′ATGCCGAGATTCTGCTACAGT3′, and reverse 5′TCCAGCGAGAGGTCGAGTTT3′, Ucp2 forward 5′ATGGTTGGTTTCAAGGCCACA3′, and reverse 5′CGGTATCCAGAGGGAAAGTGAT3′

### Animals and feeding experiments

All animal experiments were approved by the Institutional Animal Care and Use Committee (IACUC) of Rutgers the State University of New Jersey (protocol 999900492). All experiments were performed in compliance accordance with the with the ARRIVE guidelines and in accordance to IACUC regulations which meet recommendations in the Guide for the Care and Use of Laboratory Animals of the National Institutes of Health, and Public Health Service (PHS) Policy on Humane Care and Use of Laboratory Animals (Department of Agriculture, Animal Welfare Act).

Previous studies detected the difference between levels of THRB2 expression in male and female^3^. Also, to avoid effect of cyclic changes during ovarian cycle, only males were used in this study. All mice had C57BL/6J genetic background and were maintained on a standard diet (5008, LabDiet; MO, USA) with water ad libitum in a temperature-controlled facility with a 12 h light /12 h dark schedule. For HFD experiments, mice were transferred to Rodent diet 60% kcal/fat (D12492i, Research Diets, NJ, USA) at 5 weeks old and maintained on a diet as indicated. Food intake and BW were measured weekly. Unless otherwise indicated, 3.0–4.5 months old adult males were used. *Thrb2*^*WT*^ and homozygous *Thrb*^*em1Few*^ (previously called *Thrb2*^*S101A*^) mutant animals^12^ of the same age (n = 4–8) were handled daily for 7–10 days before experiment, body weight and basal food intake were measured manually as described. 1–2 animal from each group showing irregular food intake, < 2 g/day, or > 4 g/day were excluded from studies. Unless otherwise indicated, animals were fasted for 2 h to synchronize feeding, injected i.p. with vehicle, food intake were measured for 10 h with 2 h intervals, and every 24 h. After 4–7 days recovery, same animals were injected with a drug of interest. In the case the group of mice were to receive another drug, vehicle treatment were repeated. Despite of differences in food intake between individual animals, feeding behavior of each mice receiving either vehicle injection were consistently similar for at least 4 week period. Experiment with each substance were repeated at least two times. If the drug was administrated to animals previously treated with another drug, in the next group of mice the order of substance administration was changed.

Melanotan (MTII, 2 mg/kg BW, Sigma-Aldrich, MO, USA), rat Ghrelin (0.4 mg/kg, Tocris/ Biotechne, MN, USA), recombinant mouse Leptin (2 mg/kg BW, R&D Systems/ Biotechne, MN, USA) were dissolved in DPBS and injected i.p. at indicated concentration. Water-insoluble drugs were dissolved in DMSO, further resuspended with Kolliphor (Sigma-Aldrich, MO, USA) and mixed with PBS (0.1 M Phosphate buffer saline, pH 7.4) ACEA (1 mg/kg BW Tocris/ Biotechne, MN, USA), AM251 (0.6 mg/kg and 2.0 mg/kg BW, Tocris/ Biotechne, MN, USA), Trolox (20 mg/kg BW, R&D Systems, MN, USA) Naloxone (7.5 mg/kg BW, Sigma-Aldrich, MO, USA) were administrated with 2% DMSO, 10% Kolliphor in saline. 2% DMSO, 10% Kolliphor in saline was used as a vehicle for these experiments.

### Animal tissue collection and serum preparation

For hypothalamic tissue collection, at conditions indicated (fasted, refed, HFD, etc.) mice were euthanized by CO_2_ inhalation, decapitated, trunk blood collected, and tissue surrounding Arcuate nucleus (Mediobasal Hypothalamus) was collected using stainless steel brain matrix and snap-frozen. Blood was clotted for 40 min at room temperature, serum separated by centrifugation, aliquoted and frozen at − 80 °C until analysis.

### T_3_ administration and T_3_ measurements

Animals were transferred to drinking vehicle water (0.0015% BSA, 1:3000 PBS) for 6 days, and then to T_3_ water (0.0015% BSA, 1:3000 PBS, T_3_ 0.3 ug/ml, Sigma, MO, USA). Food intake and body weight was measured every two days, starting 4 days before the first transfer. Animals were euthanized by CO_2_ inhalation after 20 days on T_3_ water, tissues and blood serum were collected_._ Free serum T_3_ (free triiodothyronine, fT_3_), was measured using mouse free T_3_ ELISA Kit (Aviva Systems Biology, CA, USA) according to manufacturer protocol.

### DHE injection, Immunofluorescence, and image acquisition

Tail vein injection of dihydroethidium (*DHE*, Invitrogen, OR USA, 7 mg/kg BW, dissolved in 5% DMSO, 5% Kolliphor in PBS) was conducted two hours prior to perfusion. All animals underwent 2-week acclimation period of handling and restraint before the experiment.

WT, *Thrb*^*em1Few*^ and *Thrb*^*em2Few*^ male mice (14–16 weeks free fed, fasted and refed, or HFD animals 14–17, and 25–28 weeks old) were perfused, and brain tissues were processed as previously described^55^. Briefly, animals were anesthetized with ketamine and xylazine (100/10 mg/kg of body weight, Henry Schein Inc.; Melville, NY, USA). Intracardiac perfusion was performed with PBS (0.1 M Phosphate buffer saline, pH 7.4) followed by tissue fixation in 4% paraformaldehyde. Brains were post-fixed in 4% paraformaldehyde, at 4 °C, overnight, transferred to a 30% sucrose solution and kept at 4 °C until saturated (approximately 48 h later). Frozen brains were sectioned into 30 µm sections using a Cryostat (CM1850 UV, Leica Biosystems, IL, USA) and stored at − 20 °C in antifreeze cryoprotectant. Immunofluorescence on free floating brain sections was performed as previously described with following adjustments^56,57^. Briefly, 30 µm brain sections were washed with water, and placed in the tube containing 10 mM citric acid—sodium citrate buffer pH6 into gradually cooling water bath starting at 80 °C for 20 min, then cooling at RT for 10 min. After washing with PBS and sections were blocked in PBST containing 5% normal donkey serum, 1% BSA, and 0.5% Triton X-100 at room temperature for 2 h and incubated with PBST 5% donkey serum, 1% BSA and primary antibodies at 4 °C overnight. Tissues were washed with PBST 3 times and incubated with secondary antibody for two hours. After washing with PBST three times, brain sections were mounted onto slides using DAPI Fluoromount-G (Southern Biotech, AL, USA). Every staining batch included experimental and control sections, along with sections from previously stained animals for comparison. DHE-injected animals were stained immediately after sectioning, sections were stained with anti-POMC antibody as described, excluding citrate treatment and Triton X-100 from the protocol.

Primary antibodies: rabbit anti-HA (C29F4, 1:250, Cell Signaling, MA, USA), rabbit anti-POMC (1:1000, Phoenix Pharmaceuticals, CA, USA), sheep anti-α-MSH (1:200, EMD Millipore, Germany), rabbit anti-β-endorphin (1:200, Novus Biologicals/ Biotechne, MN, USA), goat anti-AgRP (1:200, AF634, R&D Systems, MN, USA). Conjugated antibody, anti-HA-Fluorescein (3F10 1:100, Roche, Diagnostics, Germany), anti-c-FOS Alexa Fluor 488 (1:100, Santa Cruz Biotechnology, CA, USA). Secondary antibodies Donkey anti-rabbit IgG Alexa Fluor 647, Donkey anti-rabbit IgG Alexa Fluor 488, Donkey anti-sheep IgG Alexa Fluor 555, Donkey anti-goat Alexa Fluor 647 secondary antibody (1:1000, Invitrogen, OR USA).

Six to eight hypothalamic arcuate sections per animal were scanned with a Zeiss 700 Laser Scanning Microscope (LSM700) using a Plan-Apochromat × 20 objective. Average intensity measurement and image processing were performed using Zen 2.3 software (Zeiss, Germany). For POMC neurons, DHE, POMC, β-EP, α-MSH fluorescence intensity was measured in 100–350 cells per animal, and normalized to the background within the same section. Cells were outlined in POMC channel, in blinded manner, then the other channel (i.e. DHE) was assessed, when co-stained for c-FOS, the outlined cells were counted for nuclear c-FOS presence. To calculate “POMC/Arc ROS” ratio, DHE fluorescent intensity within POMC neurons was divided by the intensity of DHE-positive POMC-negative cells within each section (background subtracted). For POMC neuron numbers, POMC-positive cells were counted in 7–9 animals (> 150 coronal sections total) per group. Average count within ~ 0.1 mm step coronal sections of Arcuate nucleus (bregma − 1.34, − 2.46) of individual animals was used for graphing.

### Statistics

Graphs and statistical analyses were performed using GraphPad Prism (San Diego, CA, USA). All values are reported as the mean ± standard error of the mean. Two-way ANOVA, Tukey’s multiple comparisons test and Sidak’s post test for repeated measures. Student *t*-test was used for comparing two groups. Statistical tests used for each experiment are indicated in figure legends, numerical *p* values are summarized in Supplementary Tables 1 and 2.

### Supplementary Information

Supplementary Information.

## Data Availability

All data generated or analyzed during this study are included in this published article [and its supplementary information files]. All additional and row data used and/or analyzed during the current study available from the corresponding author on reasonable request.
