# Supplementary material for: Regulation of hypothalamic reactive oxygen species and feeding behavior by phosphorylation of the beta 2 thyroid hormone receptor isoform
Source: Sci Rep. 2024 Mar 26;14:7200. doi: 10.1038/s41598-024-57364-9 (PMC10965981; doi:10.1038/s41598-024-57364-9)
Supplement: Supplementary file 1 — Supplementary Information. [file 41598_2024_57364_MOESM1_ESM.pdf]

Regulation of hypothalamic reactive oxygen species and feeding behavior by phosphorylation of the beta 2 thyroid hormone receptor isoform

Svetlana Minakhina[1,2*], Sun Young Kim[1], Fredric E. Wondisford[1,3*]

Supplementary material

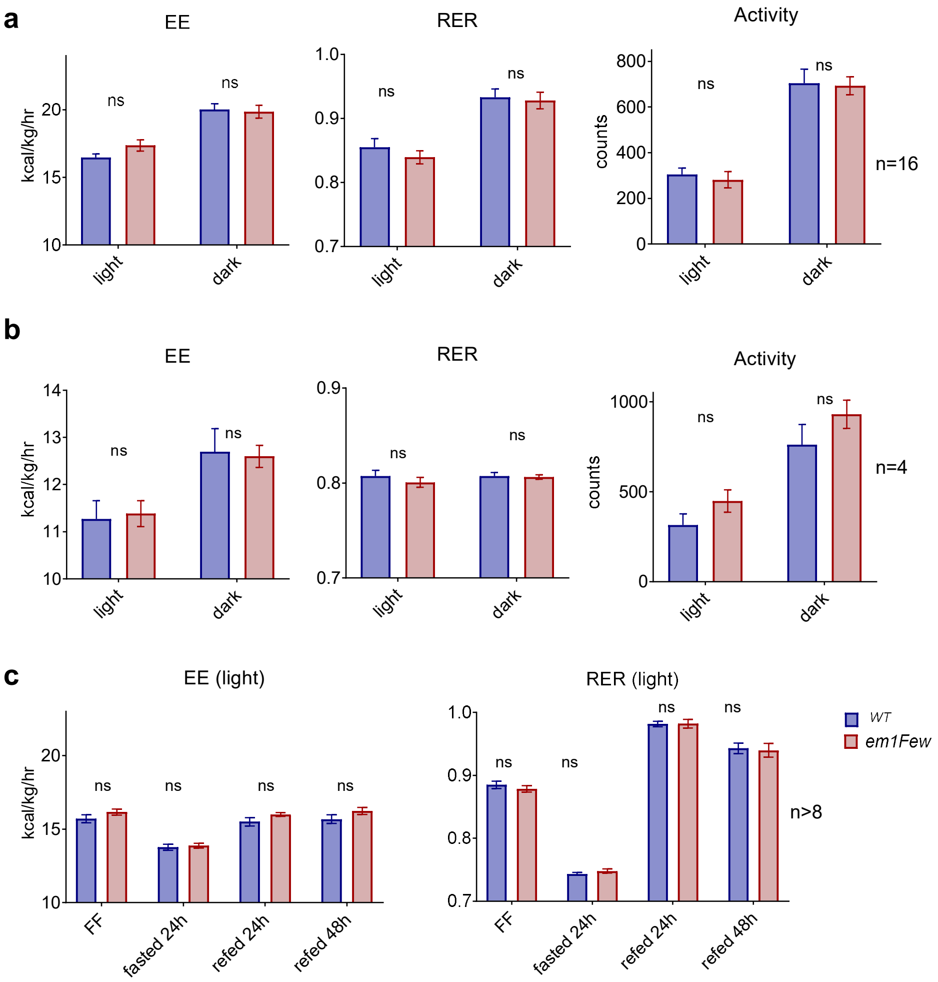

Supplementary Figure 1. S101A mutation in *Thrb2* (*Thrb*^{em1Few}) does not affect energy metabolism.

(**a**) No difference observed in Energy Expenditure (EE), Respiratory Exchange Rate (RER) or activity in WT (blue) and *Thrb*^{em1Few} (*em1Few,* pink) animals on RD (**a**), on HFD (**b**), or during fasting-refeeding (**c,** RD). Two way ANOVA and Sidak's multiple comparison tests used for statistical analysis, ns - not significant.

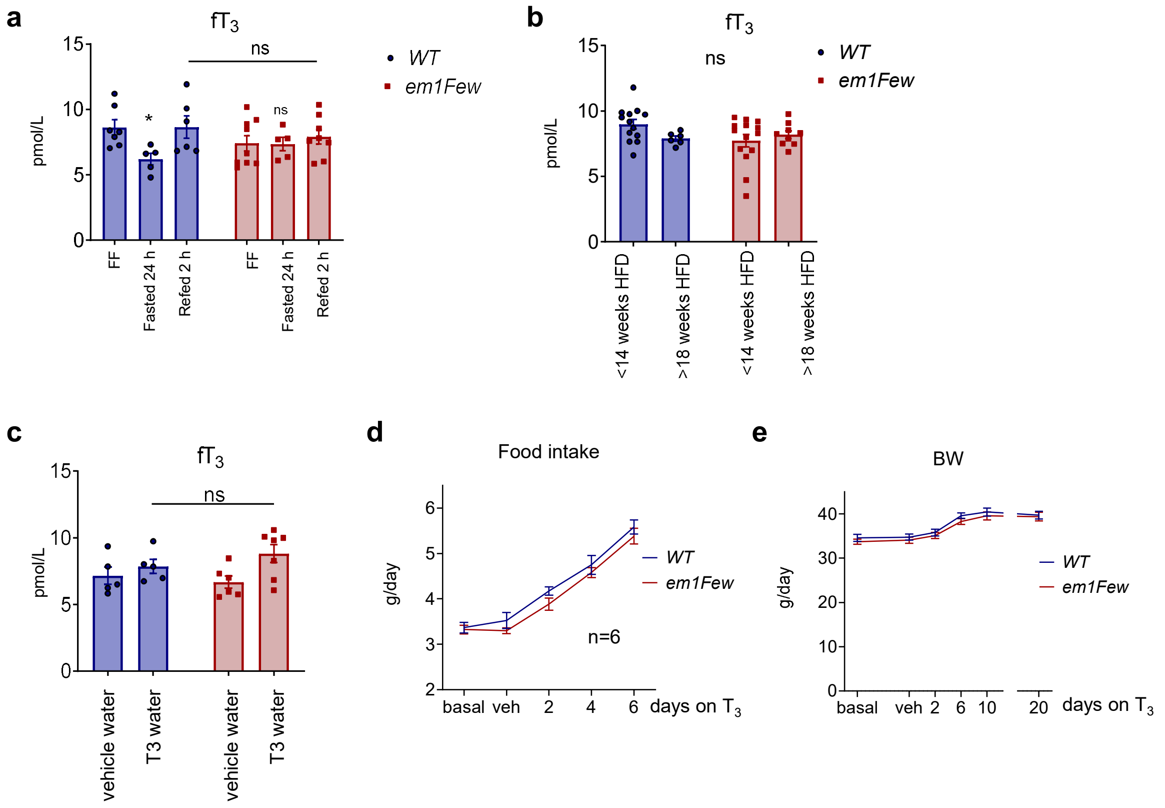

Supplementary Figure 2. Feeding phenotypes of *Thrb2*$^{S101A/S101A}$ animals are not associated with hyperthyroidism.

(**a, b**) At refeeding and HFD, serum fT_3 of *Thrb*^{em1Few} (*em1Few,* pink) animals is similar to that of WT (blue). (**c**) T_3 administration in drinking water (0.3 mg/ml) causes non-significant increase in serum fT_3 in *Thrb*^{em1Few} animals, and a similar increase in food intake (**d**) and BW (**e**) in WT and *Thrb*^{em1Few} animals. Two-way ANOVA and Sidak's multiple comparison tests used for statistical analysis, ns- not significant.

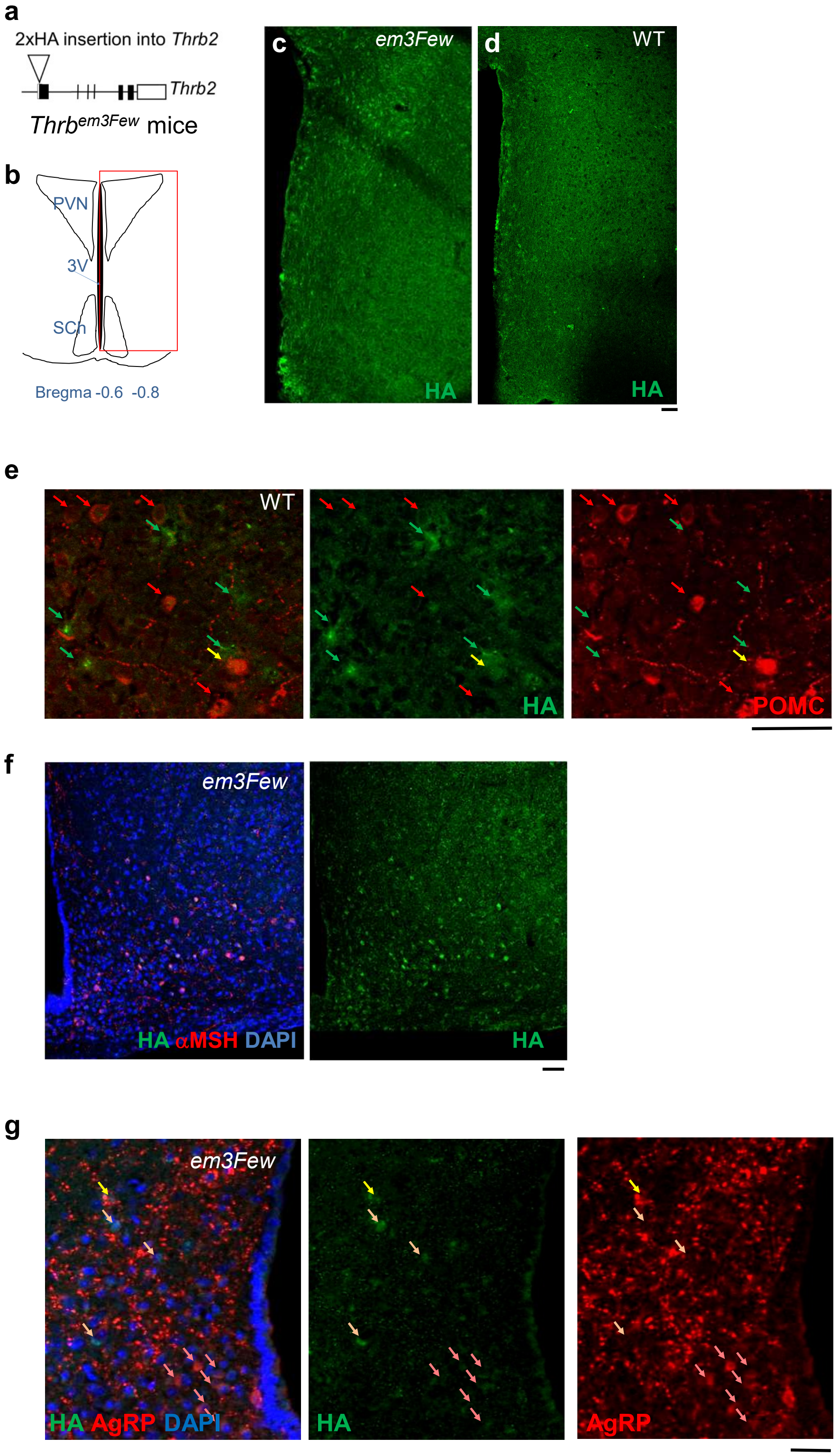

Supplementary Figure 3. HA-THRB2 expression in hypothalamus.

(**a**) Structure of endogenously tagged *HA-Thrb2 (Thrb*^{em3Few} mice*)*. (**b-d)** HA immunoreactivity is present in PVN area of Arc of *Thrb*^{em3Few} (*em3Few,* **c**) but not WT animals (**d**, rat anti-HA). (**e**) Background HA-immunofluorescence (green) in Arc and POMC neurons (red) (red arrows - POMC neurons, green arrows-.HA background, yellow arrows rare colocalization of HA and POMC staining (see Fig. 2d) (**f**) *Thrb*^{em3Few} animals exhibit co-localization of a-MSH (red) staining and HA-immunofluorescence (green, rabbit anti-HA) in Arc cross section. DNA is stained with DAPI (blue). Scale bar 50 μM. (**g**) HA-THRB2 (green, green arrows) is not found in AgRP/NPY (red) neurons. Note that AgRP/NPY neuron bodies (pink arrows) have weaker staining then AgRP neuronal projections and synaptic junctions. Some of HA-positive cells are surrounded by intense AgRP staining, likely synaptic junctions (yellow arrow), but no significant co-localization between HA and AgRP was observed in *Thrb*^{em3Few} Arc.

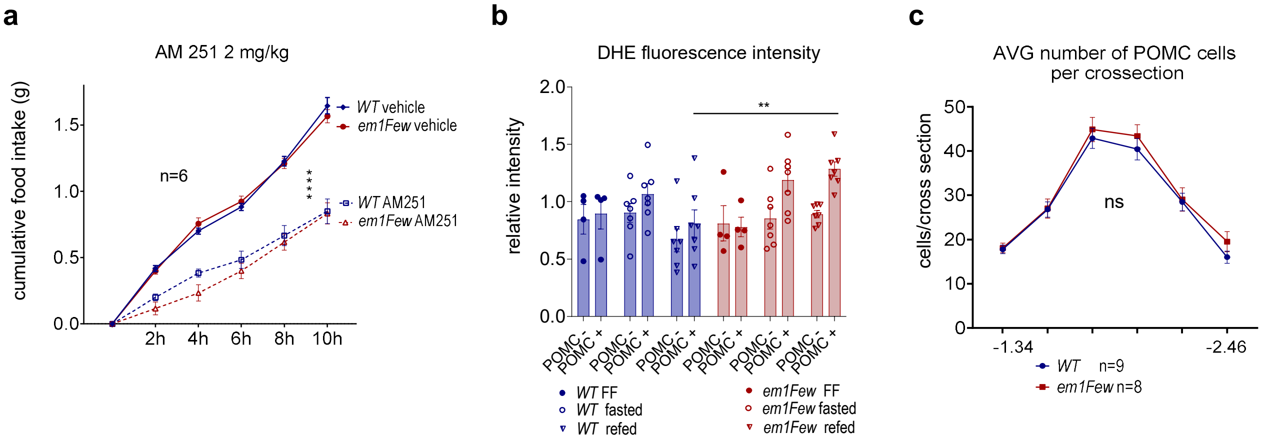

Supplementary Figure 4. Additional phenotypes of *Thrb*^{em1Few} animals.

(a) Cumulative food intake after 2 h fasting followed by AM251 injection (2 mg/kg BW, dashed lines). Solid lines show food intake of the same group of animals after vehicle injection. Food accumulation was measured every 2 h for 10 h. (**b**) The graph shows variable ROS (DHE) fluorescence intensity in POMC- and POMC+ Arc neurons in WT and *Thrb*^{em1Few} mice (*em1Few*) animals at FF, fasted, and refed states. Each dot on the graphs represents averaged measurement from ~100 cells from individual mice (**c**) Distribution of POMC neurons throughout Arc (bregma -1.34 to -2.46) in WT and *Thrb*^{em1Few} mice <14 weeks fed a RD. Each dot on the represents average count from 2-4 csos-sections of individual mice, all values are +/- SEM,

Two way ANOVA and Tukey's multiple comparison tests used for statistical analysis, ns- not significant. p values for (**a**) between vehicle and AM251 in WT animals at 4,6,8, and 10 hours <0.0001 (****) and between vehicle and AM251 in mutant animals at 4,6,8, and 10 hours <0.0001 (****). (**b**) p=0.0057 (**). Area under the curve (AUC) and unpaired t test was used for (**c**), ns (p=0.6) for not significant.

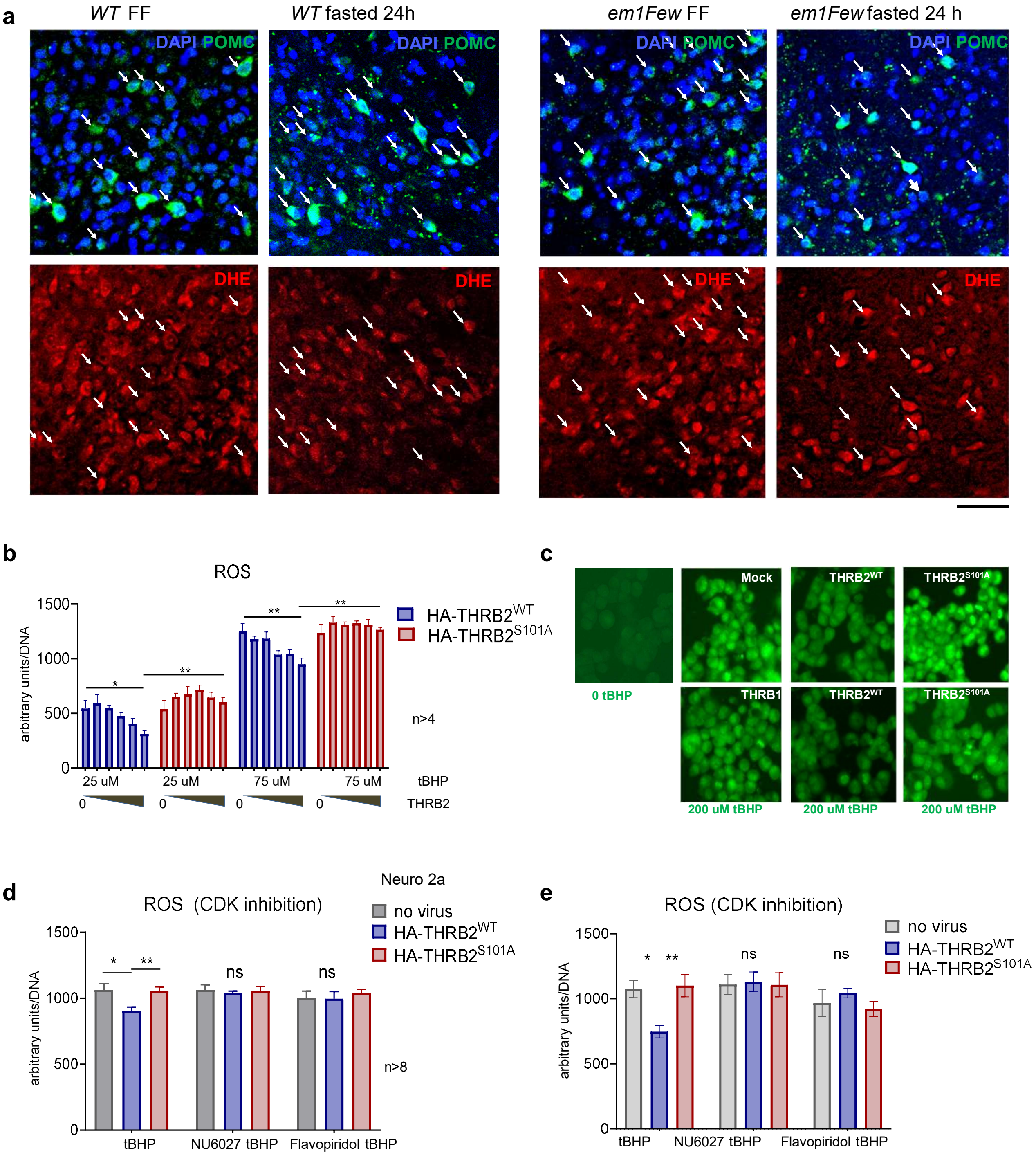

Supplementary Figure 5. ROS accumulation in POMC neurons and cultured neuroblastoma cells. (**a**) Representative images of ROS (DHE, red) accumulation in POMC (green) arcuate neurons (arrows) in FF and fasted, WT and *Thrb*^{em1Few} (*em1Few*) animals. DNA stained with DAPI (blue). Scale bar 50 μM. (**b**) Expression of incrementing amounts of HA-THRB2^{WT}, but not HA-THRB2^{S101A} reduces ROS accumulation in Neuro-2a cells in response to 25μM and 75μM tBHP treatments (2h). (**c**) Similarly, reduced ROS accumulation (CellROX^{TM} Green) is observed in N1E-115 cells expressing HA-THRB2^{WT}, but not HA-THRB1 and HA-THRB2^{S101A} treated with 200 μM tBHP for 1.5h. No ROS accumulation is observed in the absence of tBHP (0 tBHP). In both cell lines, Neuro-2a (**d**) and N1E-115 (**e**), protective effect of THRB2^{WT} is abolished by treatments with NU6027 or Flavopiridol. Two way ANOVA with Tukey's multiple comparison test was used for statistical analysis. All values are +/- SEM, (**b**) *p=0.0467, **p= 0.003, 0.0065, and 0.0024 (left to right), no significant difference were found within groups of cells expressing HA-THRB2^{S101A}; (**d**) *p=0.0188, **p=0.0023, (**e**) *p=0.0125, **p=0.0043, ns – no significant difference were found within groups treated with NU6027 or Flavopiridol.

a

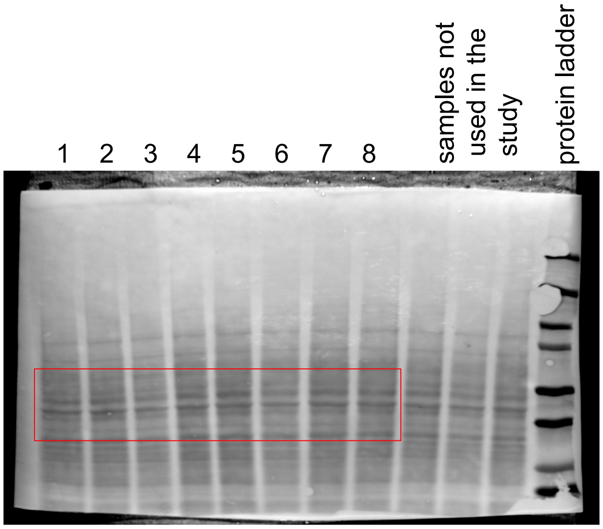

b

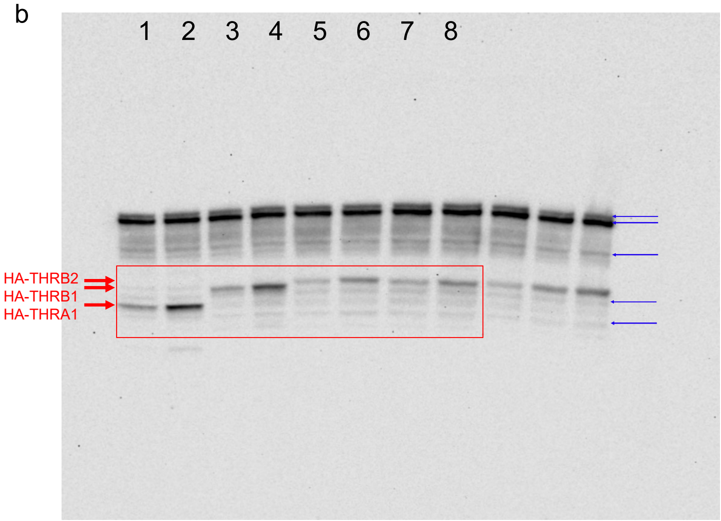

Supplementary Figure 6. Full size Western blot used for Figure 4f. Expression of HA-THR isoforms in Neuro2a cells.

(a) Ponceau S staining of Nitrocellulose membrane with transferred cellular proteins. Red rectangle shows fragment of the gel used in Fig. 4f. (**b**) Membrane probed with anti HA antibody (rabbit) and anti-rabbit HRP. Red rectangle shows the fragment of the gel used in Fig. 4f. Red arrows show HA-THRA1 (lanes 1, 2), HA-THRB1 (lanes 3, 4), HA-THRB2 (lanes 5, 6) and HA-THRB2^{S101A} (lanes 7, 8). Blue arrows show non-specific bands present in all lanes irrespective to THR isoform.

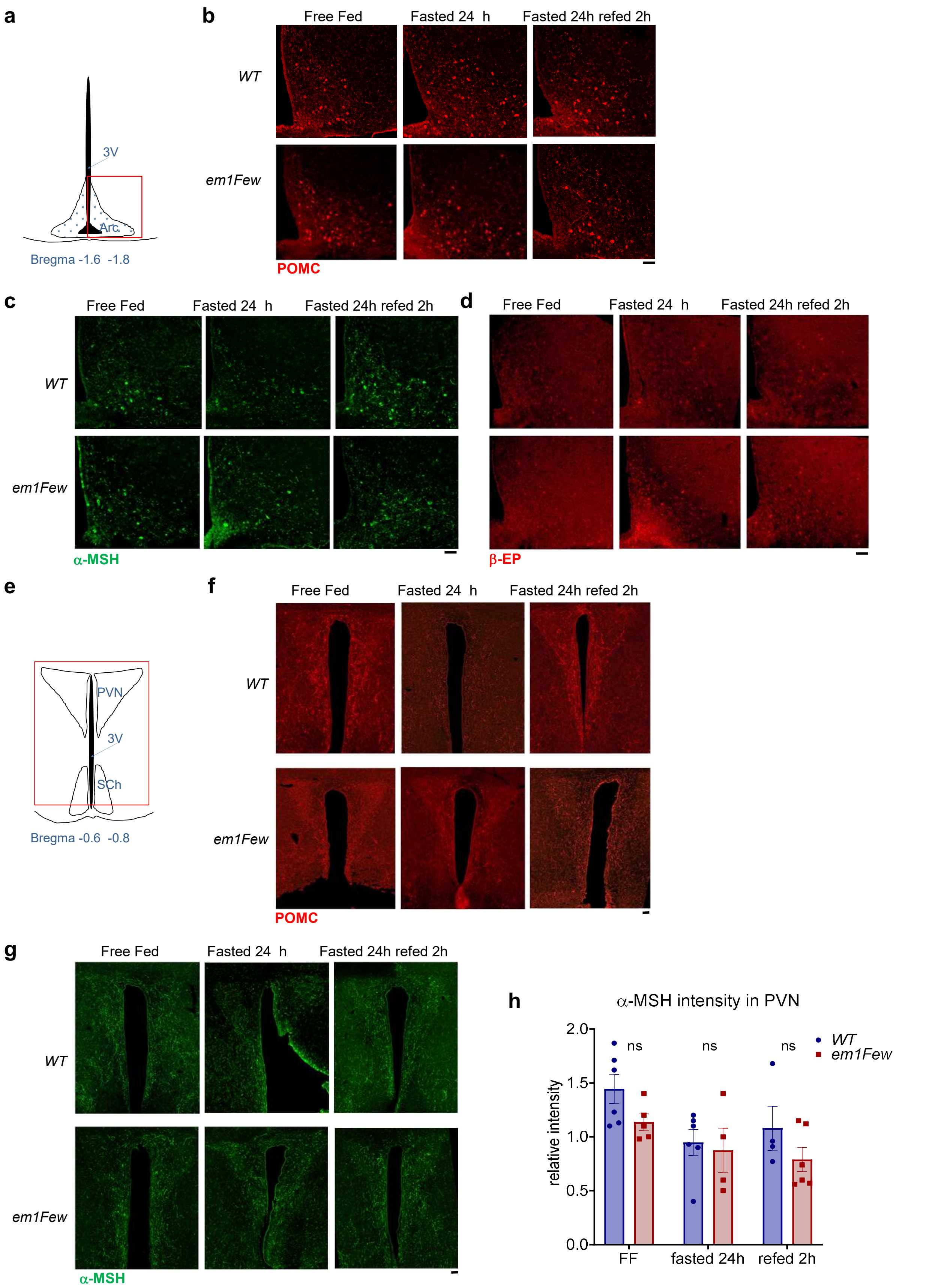

Supplementary Figure 7. Effect of *Thrb^{em1Few}* on POMC, α-MSH and β-EP production in Arc in response to fasting/refeeding.

Comparison of POMC hormones in Arc (**a-d**) and PVN (**e-h**) of WT and *Thrb^{em1Few}* (*em1Few)* animals. POMC staining (red) in Arc POMC neurons (b) and PVN (f). β-EP (red) in Arc POMC neurons (**d**). α-MSH staining (green) in Arc POMC neurons (**c**) and PVN (**g**), (**h**) relative florescent intensity in PVN.). Scale bar 50 μM. Each dot on the graphs represents averaged measurement from 2-3 PVN cross sections of individual mice, all values are +/- SEM, Two way ANOVA and Sidak's multiple comparison tests used for statistical analysis, ns for not significant.

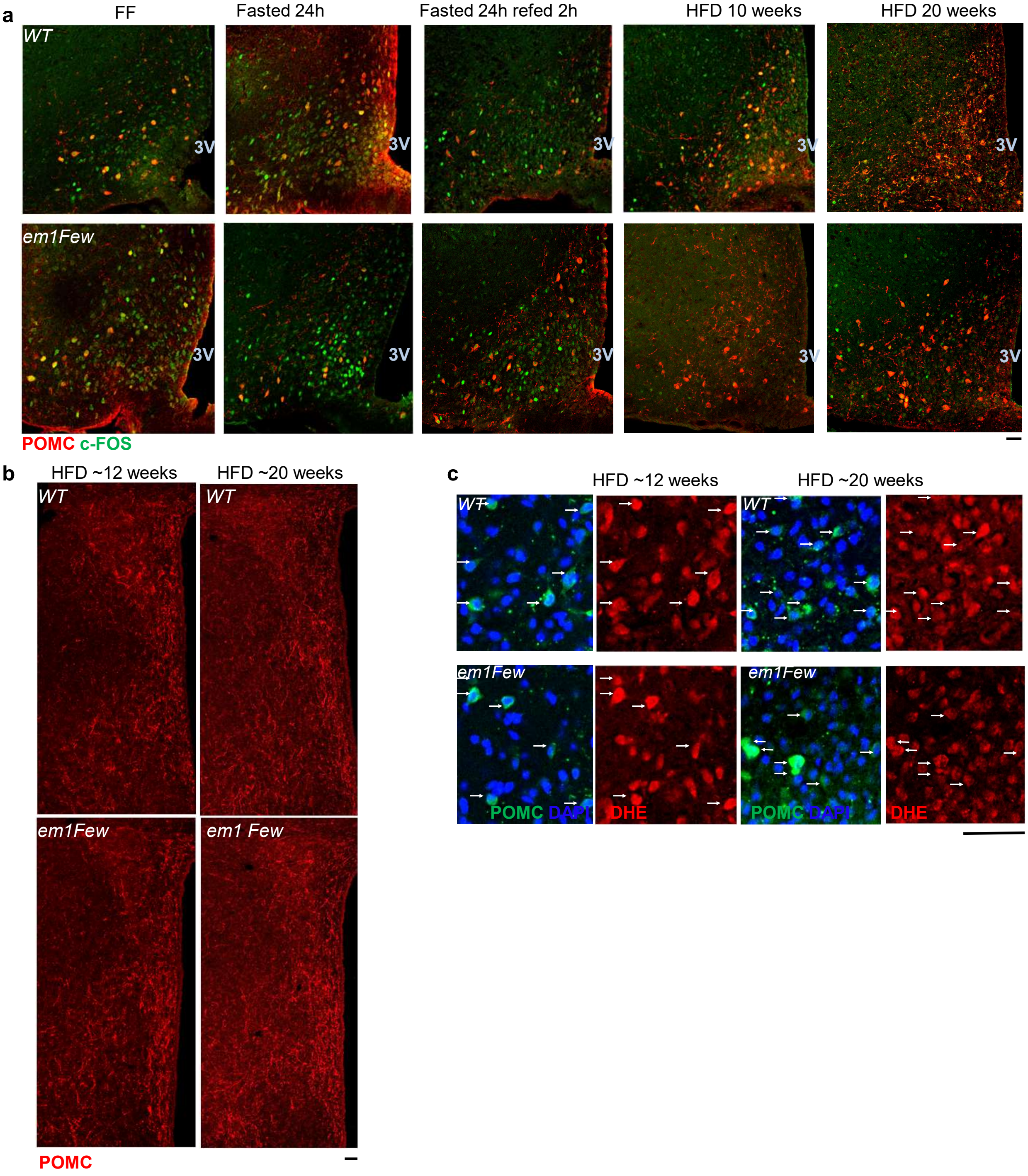

Supplementary Figure 8. Hypothalamic phenotypes in WT and *Thrb*^{em1Few} animals.

(**a**) Examples of c-FOS (green) staining in Arc of WT and *Thrb*^{em1Few} *(em1Few)* animals at RD and HFD. POMC neurons shown in red. (**b**) POMC staining (red) in PVN of WT and *Thrb*^{em1Few} animals kept on HFD for 14 and 20 weeks. (**c**) Representative images of WT and *Thrb*^{em1Few} mutant Arc, POMC (green) neurons with ROS (DHE, red) accumulation (arrows). DNA is stained with DAPI (blue). Scale bar 50 µM.

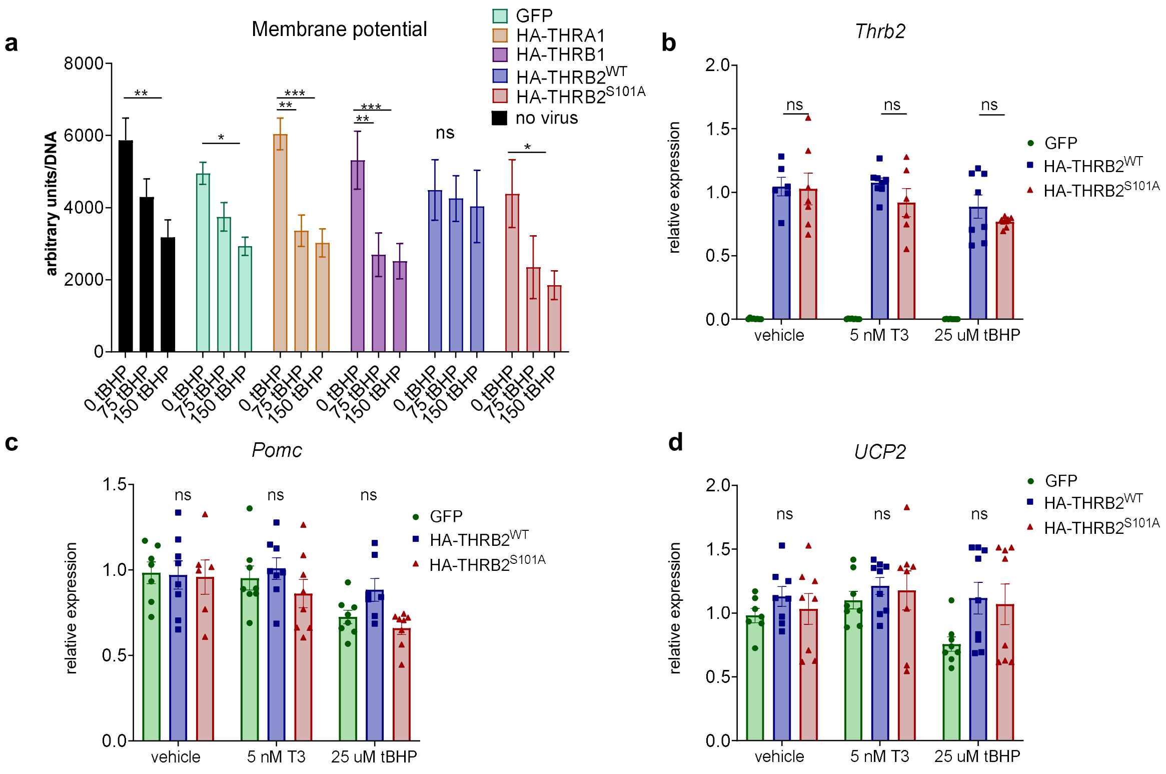

Supplementary Figure 9. Effects of pTHRB2 in Neuro2a cells.

(**a**) Membrane potential, an early marker for cell death, was measured in Neuro-2a cells expressing GFP, HA-THRA1, HA-THRB1, HA-THRB2^{WT} and HA-THRB2^{S101A} using MitoTracker Red CMXRos. tBHP treatment at 75 μM and 150 μM performed for 2 h. Representative bar graph from 3 similar experiments is shown, n>5, values are mean +/- SEM. Two way ANOVA with Tukey's multiple comparison test was used for statistical analysis ***p<0.001, **p<0.01,*p<0.05, ns for not significant. (p values left to right 0.0095, 0.0342, 0.0003, 0.0018, 0.0009, 0.003, 0.0192) (**b-d**) qRT-PCR bar graphs show no difference in *Pomc* (**c**) or *Ucp2* (**d**) expression in Neuro2a cells expressing GFP, HA-THRB2^{WT} and HA-THRB2^{S101A} at similar levels (**b**). Expression levels were normalized to *RpL13a*. Cells were treated with 25 μM tBHP or 5 nM T_3 for 4 hours. Independent replicates shown by dots on the graphs, all values are +/- SEM, ns for not significant. Two way ANOVA and Tukey's multiple comparison test was used for statistical analysis.

**Figure 1**	p values	**Figure 3**	p values	**Figure 3**	p values	**Figure 4**	p values	**Figure 6**	p values
**1(a)**	0.015	**3(a)**	<0.0001	**3(f)**	0.0014	**4(a)**	0.2574	**6(a)**	0.0007
	0.008		<0.0001		0.0009		0.0032		<0.0001
	0.029	**3(b)**	0.002		0.0023	**4(d)**	0.0023		0.0178
**1(b)**	0.015		0.0033		<0.0001		0.0028	**6(b)**	0.0029
**1(c)**	0.035	**3(c)**	<0.0001	**3(g)**	0.0104	**4(e)**	0.0012		0.0445
	0.042		0.0128		0.0013	**4 (f)**	0.0005	**6(f)**	<0.0001
	0.034		0.0005		0.0012	**Figure 5**	p values		0.0271
	0.003		0.0042		0.0015	**5(b)**	0.0013	**Figure 6**	AUC
	0.002	**3(d)**	<0.0001	**3(h)**	0.0016		0.0001	**6(i)**	232.6
	0.031		<0.0001		0.0002		0.0035		216.7
**1(d)**	0.0001	**3(e)**	0.0016		<0.0001	**5(c)** ns	0.359		p value
	<0.0001	ns	0.7526		<0.0001		0.0314	ns	0.4768
**1(e)**	<0.0001		<0.0001	**3(i)**	0.563	ns	0.3563	**6(j)**	252.4
	0.0005	ns	0.385		0.1022	**5(d)**	0.0012		169
	<0.0001		<0.0001	**3(j)**	0.03	ns	0.19		p value
		ns	0.0738	**3(k)**	0.0004		0.019		0.0003
**Figure 2**	p value		<0.0001		0.035	**5(f)**	<0.0001		
**2(d)**	<0.0001		<0.0001		0.0085	**5(i)**	0.0024		
	<0.0001		<0.0001		0.0251		0.0134		

Supplementary Table 1. p-values for Figures 1-6, as calculated in GraphPad Prism 10, in order they appear on each figure panel (left to right), statistical test used are indicated in each figure legend. Area under the curve (AUC) and non-pared t-test, p value is calculated for Fig. 6i and j.

genotype/single drug treatment	3(a) MTII		3(c) Ghrelin		3(e) Leptin		3(g) ACEA		3(h) AM251	
		p value		p value		p value		p value		p value
2h										
WT vehicle vs. S101A vehicle	ns	0.639	ns	0.925	ns	0.979	ns	0.874	ns	0.989
WT vehicle vs. WT treated	*	0.044	ns	0.556	ns	0.082	ns	0.232	ns	0.132
S101A vehicle vs. S101A treated	ns	0.17	ns	0.795	ns	0.631	**	0.009	ns	0.88
WT treated vs. S101A treated	ns	0.934	ns	0.809	ns	0.856	ns	0.141	ns	0.059
4h										
WT vehicle vs. S101A vehicle	ns	0.811	ns	0.997	ns	0.631	ns	1	ns	0.766
WT vehicle vs. WT treated	**	0.008	**	0.005	**	0.002	ns	0.699	**	0.002
S101A vehicle vs. S101A treated	**	0.003	*	0.022	ns	0.753	***	4E-04	ns	0.343
WT treated vs. S101A treated	ns	0.639	ns	0.985	ns	0.444	*	0.01	*	0.035
6h										
WT vehicle vs. S101A vehicle	ns	0.934	ns	0.985	ns	0.505	ns	0.93	ns	0.894
WT vehicle vs. WT treated	**	0.001	***	5E-04	****	<0.0001	ns	0.669	***	2E-04
S101A vehicle vs. S101A treated	*	0.044	ns	0.056	ns	0.385	***	3E-04	ns	0.682
WT treated vs. S101A treated	ns	0.934	ns	0.848	ns	0.33	**	0.001	**	0.003
8h										
WT vehicle vs. S101A vehicle	ns	0.291	ns	0.809	ns	0.534	ns	0.43	ns	0.992
WT vehicle vs. WT treated	****	<0.0001	****	<0.0001	****	<0.0001	ns	0.407	****	<0.0001
S101A vehicle vs. S101A treated	****	<0.0001	*	0.013	ns	0.074	**	0.002	ns	0.682
WT treated vs. S101A treated	ns	>0.9999	ns	0.327	ns	0.263	**	0.001	****	<0.0001
10h										
WT vehicle vs. S101A vehicle	ns	0.639	ns	0.98	ns	0.631	ns	0.714	ns	0.514
WT vehicle vs. WT treated	****	<0.0001	***	5E-04	****	<0.0001	ns	>0.9999	****	<0.0001
S101A vehicle vs. S101A treated	****	<0.0001	**	0.004	****	<0.0001	ns	0.067	ns	0.12
WT treated vs. S101A treated	ns	0.811	ns	0.997	**	0.009	**	0.002	****	<0.0001

genotype/double drug treatment	3(d) Ghrelin/ Leptin		3(j) ACEA/Trolox		3(k) ACEA/Naloxone	
		p value		p value		p value
2h						
WT single vs. S101A single	ns	0.7956	ns	0.1876	ns	0.0595
WT single vs. WT double	ns	0.4284	ns	0.9682	ns	0.6872
S101A single vs. S101A double	ns	0.434	ns	0.3759	***	0.0004
WT double vs. S101A double	ns	0.7045	ns	0.9997	ns	0.9427
4h						
WT single vs. S101A single	ns	0.984	*	0.0193	**	0.0023
WT single vs. WT double	**	0.0098	ns	0.97	ns	0.7664
S101A single vs. S101A double	**	0.003	ns	0.1285	*	0.035
WT double vs. S101A double	ns	0.6802	ns	0.9954	ns	0.3312
6h						
WT single vs. S101A single	ns	0.8364	**	0.003	****	<0.0001
WT single vs. WT double	****	<0.0001	ns	0.5301	ns	>0.9999
S101A single vs. S101A double	***	0.0002	*	0.03	**	0.0085
WT double vs. S101A double	ns	0.9193	ns	0.9058	ns	0.5149
8h						
WT single vs. S101A single	ns	0.3023	**	0.0028	****	<0.0001
WT single vs. WT double	****	<0.0001	ns	0.6406	ns	0.566
S101A single vs. S101A double	****	<0.0001	ns	0.0802	*	0.0251
WT double vs. S101A double	ns	0.9985	ns	0.9995	ns	0.4073
10h						
WT single vs. S101A single	ns	0.9972	**	0.0035	****	<0.0001
WT single vs. WT double	****	<0.0001	ns	0.2882	ns	0.7114
S101A single vs. S101A double	****	<0.0001	ns	0.4776	ns	0.6397
WT double vs. S101A double	ns	0.9994	ns	0.998	*	0.0234

genotype/treatment	AUC	AUC	AUC	AUC	AUC
WT vehicle	4.11	4.72	3.977	4.489	4.05
S101A vehicle	3.77	4.8	3.453	4.818	4.072
WT treatment	2.73	6.81	2.5	5.006	2.905
S101A treatment	2.68	6.338	2.851	6.317	3.817
**AUC comparison**	**p value**	**p value**	**p value**	**p value**	**p value**
WT vehicle vs. WT treated	<0.0001	3E-04	<0.0001	0.343	<0.0001
S101A vehicle vs. S101A treated	3E-04	0.007	<0.0001	0.01	0.009
WT vehicle vs. S101A vehicle	0.169	0.864	<0.0001	0.552	0.802
WT treated vs. S101A treated	0.835	0.321	0.002	0.02	<0.0001

genotype/treatment	AUC	AUC	AUC
WT single	6.81	5.006	5.316
S101A single	6.338	6.317	6.439
WT double	4.22	5.429	5.305
S101A double	3.838	5.373	5.632
**AUC comparison**	**p value**	**p value**	**p value**
WT single vs. WT double	<0.0001	0.011	0.9761
S101A single vs. S101A double	<0.0001	<0.0001	0.0373
WT single vs. S101A single	0.2938	<0.0001	0.0038
WT double vs. S101A double	0.3922	0.7242	0.3827

Supplementary Table 2. p values associated with Figure 3. Results of two way ANOVA, Tukey's multiple comparison test with numerical p values performed in GraphPad Prism 10. Single drug treatments vs vehicle on the left, double drug treatments vs single drug treatment on the right. Comparisons of Area under the curve (AUCs) are on the bottom.
